# Physics-Informed Deep Learning Surrogate for Mechanical-Response Prediction and Failure Assessment of High-Performance Polymer Composites

**DOI:** 10.3390/polym18212678

**Published:** 2026-10-28

**Authors:** Md Ehtesam Haque, Ramasamy Naidu, Md Arifuzzaman, Ayed Eid Alluqmani, Muhammad Ali Martuza, Akm Azad

**Affiliations:** 1 Department of Computer Science, College of Computer Science and Information Technology (CCSIT), King Faisal University, Al-Ahsa 31982, Saudi Arabia; 2 Department of Computer Engineering, College of Computer Science and Information Technology CCSIT, King Faisal University, Al Ahsa 31982, Saudi Arabia; 3 Department of Civil and Environmental Engineering, College of Engineering, King Faisal University, Al-Ahsa 31982, Saudi Arabia; 4 Department of Civil Engineering, Faculty of Engineering, Islamic University of Madinah, Al-Madinah Al-Munawara, Prince Naif Ibn Abdulaziz, Al Jamiah, Medina 42351, Saudi Arabia; 5 Department of Computer Engineering, College of Computer, Qassim University, Buraydah 51452, Saudi Arabia; 6 Department of Mathematics and Statistics, Faculty of Science, Imam Mohammad Ibn Saud Islamic University (IMSIU), Riyadh 13318, Saudi Arabia

**Keywords:** polymer composites, surrogate modeling, physics-informed machine learning, deep belief network, physics-informed recurrent neural network, golden jackal optimization, failure assessment

## Abstract

Polymer composites show complex, nonlinear mechanical behavior. This makes their rational design hard. Running tests or high-fidelity simulations for every candidate design is slow and costly. Purely data-driven models are fast, but they can break basic mechanics outside the training range. Most physics-informed (PI) models, on the other hand, target a single constitutive law or failure mode, and their loss weights are set by hand. This study presents a DBN-GJO-PI-RNN surrogate that links deep feature learning, physics-constrained learning and failure screening in one workflow. A Deep Belief Network (DBN) learns compact latent features from the material and loading inputs. A PI Recurrent Neural Network (PI-RNN) then predicts the mechanical response under a composite loss with data, constitutive, equilibrium and boundary terms. Golden Jackal Optimization (GJO) tunes the network settings and the three loss weights together. Its fitness function rewards both accuracy and physical consistency. The predicted failure index is then checked against the onset criterion (FI ≥ 1). We tested the framework on a public dataset of 5000 polymer-composite samples with six response targets. On the test set, it reached a MAE of 1.1132, a RMSE of 1.3962 and an R^2^ of 0.9932. Its RMSE was 38.3% lower than that of the best purely data-driven baseline (TCN) and 50.6% lower than that of a standard RNN. The ablation study showed that each module reduced the error. The framework offers a fast, physics-aware surrogate for screening polymer-composite designs before costly tests or simulations.

## 1. Introduction

Polymer composite materials with high specific strength, stiffness, light weight, anti-corrosion properties, and flexible designs have been developed as critical engineering materials because of these properties [1]. These materials are utilized in a variety of industries, including aerospace, automotive, marine, energy, and structural industries, which require an accurate understanding of their mechanical behaviors and failure modes [2]. Flexible glass for infrared adaptive optics is another example of an advanced material where its mechanical behavior matters in use [3]. The heterogeneity, anisotropy, and nonlinearity of these materials make it difficult to understand their properties’ relationship with loading conditions, stresses, strains, and damage progression [4]. Experiments are required to characterize these materials, but the processes are often costly, laborious, and challenging under different operating conditions [5]. Combining numerical simulation with experiments is one way to reduce this burden, as seen with the process optimization of electrolytic-assisted milling of titanium alloy [6]. Consequently, data-driven DL has received growing interest for learning the complicated behaviors of these composite materials using experimental and verified datasets [7]. Pure data-driven approaches can result in physically unreasonable predictions when the datasets and loading conditions are not sufficient [8].

The mechanical performance of high-performance polymer composite materials depends on the properties of the matrix, reinforcement materials, fiber orientation, volume fractions, and interfacial behavior [9]. Additionally, external factors including the degree of loading, loading direction, strain rates, temperatures, and cyclic loading may affect their stress–strain behavior [10]. Time-dependent effects, such as creep, also change the mechanical response of polymer-based materials, as observed for HTPB solid propellant [11]. Defects, voids, delamination, interfacial debonding, and other flaws might help initiate and propagate the damage [12]. Failure can also switch modes when geometric constraints change. A sawtooth-to-brittle transition in cubane architectures is one such example [13]. This interaction leads to nonlinear correlations between the characteristics of the material and its mechanical behavior [14]. The conventional experimental techniques can account for such behaviors, but they usually take a lot of time and money [15]. Thus, adequate computational models should be developed to predict the mechanical behavior under various material and loading conditions [16]. Mesoscale simulation is one such tool. For example, a discrete element model has been used to study the meso-structure of recycled aggregate stabilized with geopolymer [17].

Different machine learning (ML) and DL of One-Dimensional Convolutional Neural Network (1D-CNN), Long Short-Term Memory (LSTM), Bidirectional LSTM (BiLSTM), Gated Recurrent Unit (GRU), Temporal Convolutional Network (TCN), DBN, and PI Neural Network (PINN) algorithms have been studied for composite mechanical-response prediction. The models based on CNN techniques can detect local features, while LSTM, BiLSTM, and GRU can detect sequential relations in mechanical data [18]. The TCN algorithm offers an alternative way of temporal modeling using convolution techniques, and the DBN technique can learn hierarchical nonlinear feature representations [19]. PINN techniques include physical governing equations in the learning process and can provide more physical consistency than purely data-driven models. However, CNN and recurrent models traditionally focus on optimizing prediction errors and can break physical governing equations under unseen conditions [20], DBN-based models can offer effective representations without enforcing the governing mechanical equations [21], PINN-based techniques can be sensitive to the choice of loss weight and model parameters, and traditional optimization methods cannot find the right configurations efficiently [22]. Recent work applies causal inference-based neural architecture search to a PINN for fatigue life prediction [23]. This reflects the same need for a systematic search of model settings.

To address these challenges, this study proposes a DBN-GJO-PI-RNN framework, which combines the advantages of deep feature learning, PI recurrent modeling, and metaheuristic optimization for high-performance polymer composites. First, DBN is employed to capture nonlinear features based on material properties, loads, and response data; PI-RNN is used to learn nonlinear mechanical responses based on the physics constraint equation. Second, a PI loss function is defined that consists of three components: data loss, physics-residual loss, and constraint loss, thereby enhancing the accuracy and physical consistency of predictions. Third, GJO is used to optimize the PI-RNN hyperparameters to obtain an efficient PI-RNN. Finally, the framework conducts failure-criterion evaluation to analyze the failure behavior based on new composite conditions. The major contribution of our framework is the integrated combination of DBN feature extraction, PI-RNN learning, GJO-based optimization, and failure assessment. The key contributions of this research are summarized below:

⮊ Develop a novel DBN-GJO-PI-RNN framework for predictive modeling and failure assessment of high-performance polymer composites.
⮊ Integrate DBN-based hierarchical feature extraction with PI-RNN to learn nonlinear and sequential composite mechanical behavior.
⮊ Formulate a PI loss function combining data, physics, and constraint terms to enhance physical consistency.
⮊ Optimize the PI-RNN configuration using GJO to improve prediction accuracy and model robustness.
⮊ Establish an integrated strategy for mechanical-response prediction and failure assessment of high-performance polymer composites.

### 1.1. Novelty of the Study

Earlier work on polymer composites falls into two groups. Data-driven models such as DNN, CNN and LSTM learn property trends well, but they ignore mechanics [24,25,26,27]. PI models respect mechanics, but they usually solve one constitutive law or one failure mode. Their loss weights are also tuned by trial and error [28,29,30,31,32]. This study closes that gap in four ways.

First, it chains a DBN and a PI-RNN. The DBN compresses the raw material and loading inputs into a 64-dimensional latent vector. The physics terms then act on the recurrent predictions made from these features. None of the cited PI composite models used a separate unsupervised feature-learning stage of this kind.

Second, GJO tunes the physics-loss weights (λd, λp, λc) together with the network settings. Its fitness function adds the physics and constraint residuals to the validation error (Equation (21)). The search favors models that are accurate and physically consistent at the same time. This replaces the manual weight tuning that limits many PINN studies [22]. Recent PINN work searches the network architecture automatically [23]. The search here also covers the three loss weights.

Third, one surrogate predicts six mechanical responses and a failure index in a single pass. A designer can screen a composite for failure onset (FI ≥ 1) without running a separate damage model.

Fourth, a stepwise ablation shows what each module adds. PI learning cuts the MAE of a standard RNN by 20.2%. DBN features cut it by a further 20.5%, and GJO tuning by another 19.7%.

To our knowledge, no earlier study has combined these four elements for polymer composites. The result is a machine-learning surrogate that can stand in for repeated tests or simulations during early design screening.

The proposed DBN-GJO-PI-RNN framework combines deep feature learning, GJO-based hyperparameter optimization, and physics-informed recurrent prediction within a unified architecture. Unlike approaches that apply these techniques separately, it integrates their complementary functions to improve feature representation, optimization efficiency, and physics-informed prediction. This integration aims to enhance predictive accuracy and consistency in polymer-composite mechanical behavior.

### 1.2. Research Organization

This paper is organized as follows: Section 2 presents the literature review, Section 3 describes a developed DBN-GJO-PI-RNN framework, Section 4 presents an experimental setup and evaluation, Section 5 discusses the results, and Section 6 concludes the study and outlines future work.

## 2. Literature Review

Recent studies have increasingly explored DL techniques for predicting the mechanical behavior and material properties of polymer and fiber-reinforced composites. Dean et al. [28] developed a PIDL constitutive framework to predict the complex anisotropic, nonlinear, and pressure-dependent mechanical behavior of short fiber-reinforced polymers (SFRPs). The study aimed to improve the generalization of constitutive modeling under varied loading conditions by integrating physical laws with neural-network learning. The method employed LSTM networks, scalar invariants based on transverse isotropy, thermodynamic consistency, material symmetry, and synthetic data generated from a validated continuum-mechanical model; an open-hole tensile (OHT) test was used for validation. The results demonstrated improved stress prediction after retraining with randomly generated loading paths, showing the potential of PIDL for generalizable composite constitutive modeling. The initial model showed limitations under complex multiaxial stress states and required additional retraining with diverse loading paths, indicating a dependence on representative training data and limited robustness for highly complex loading conditions.

Liu et al. [33] introduced a DL framework with an attention mechanism and three-dimensional stress-state information to efficiently predict compressive mechanical properties and failure modes of embedded wrinkle thick-section composites. This objective was to reduce the computational cost of high-fidelity simulations while accurately modeling composite strength, stiffness, stress–strain behavior, and damage modes. This framework employed CNN-based strength, mechanical behavior, and failure prediction networks, with an attention-based loss function used to improve local high-damage-region prediction; training data were generated using 3D finite-element simulations with a progressive damage method. The framework accurately predicted compressive mechanical properties and failure modes and identified an influence of wrinkle parameters on composite behavior. This approach relies on high-fidelity finite-element simulations for dataset generation and primarily focuses on wrinkle-induced composite damage, limiting its general applicability to diverse polymer-composite loading and failure conditions. Bahtiri et al. [29] examined a PIDL constitutive model to predict the viscoelastic–viscoplastic behavior of short fiber-reinforced nanoparticle-filled epoxy nanocomposites under varying conditions. The framework combined LSTM and feed-forward neural networks (FNNs) to model internal variables and free-energy functions while enforcing thermodynamic consistency. Synthetic constitutive-model data and cyclic loading–unloading experimental data were used for training, achieving accurate mechanical-response prediction under different fiber, nanoparticle, and hygrothermal conditions. The study does not incorporate deep feature extraction, metaheuristic optimization, or explicit failure-criterion assessment, limiting its scope for integrated composite prediction and failure analysis.

Yao et al. [30] conducted a PIML model to predict Mode I fatigue delamination propagation considering fiber-bridging effects. The study used Mode I fatigue experiments, a Paris-type semi-empirical correlation, and PINNs to incorporate fatigue-damage evolution as a physical constraint. The PINN achieved accurate crack-growth-rate predictions and outperformed conventional ML and the Paris-type model. This framework is specifically focused on Mode I fatigue delamination and fiber-bridging effects, limiting its applicability to broader mechanical-response prediction and diverse failure mechanisms in high-performance polymer composites. Linghu et al. [31] examined a Higher-Order Multiscale PINN (HOMS-PINN) framework for efficiently solving multiscale elastic problems in composite materials with discontinuous and high-contrast properties. The objective was to reduce the computational cost and overcome the limitations of conventional DL and direct PINN approaches. The method integrated higher-order multiscale modeling, PINNs, automatic differentiation, independent neural networks, homogenization, and transfer learning to capture microscopic and macroscopic mechanical behavior. Numerical experiments on high-contrast, multi-inclusion, and three-dimensional composites demonstrated accurate multiscale predictions with reduced computational expense. It primarily addresses elastic multiscale analysis and does not explicitly consider sequential mechanical-response prediction, deep feature extraction, hyperparameter optimization, or failure assessment. Malashin et al. [24] investigated the application of DL for predicting the mechanical properties of natural-fiber polymer composites under different material and processing conditions. The objective was to accurately model nonlinear relationships between fiber type, polymer matrix, surface treatment, and processing parameters. The study used linear regression, Random Forest, Gradient Boosting, and Deep Neural Networks (DNNs), with Optuna-based hyperparameter optimization to determine the best DNN architecture. Using experimental data from flax, cotton, sisal, and hemp fibers incorporated into PLA, PP, and epoxy matrices, the optimized DNN achieved the best predictive performance. However, the study was purely data-driven and did not incorporate governing physical laws, constitutive constraints, PI loss functions, or explicit failure assessment, limiting its ability to ensure physical consistency under unseen loading conditions.

Nasri and Toubal [25] investigated the application of an Artificial Neural Network (ANN) for predicting a mechanical and impact performance of short natural-fiber thermoplastic biocomposites exposed to ultraviolet radiation. The objective was to predict tensile strength, maximum impact load, and absorbed impact energy using non-destructive experimental parameters. The study employed a MATLAB-based ANN model, with mass, hardness, roughness, and natural frequencies as input features and correlation measures and MSE for performance evaluation. The results demonstrated that ANN can effectively capture the relationship between non-destructive measurements and the mechanical behavior of natural-fiber thermoplastic composites. The ANN model primarily establishes empirical relationships between input measurements and material responses, without explicitly modeling the sequential evolution of mechanical behavior or the underlying physical mechanisms governing degradation and failure. Yin et al. [26] developed a CNN-based framework to predict stress–strain behavior and mechanical properties of fiber-reinforced composites with random microstructures. The study used Peridynamics (PD) to generate stress–strain data, Principal Component Analysis (PCA) for dimensionality reduction, and a DenseNet121-based CNN with data augmentation and MSE loss for prediction. The framework effectively captured the relationship between microstructure and mechanical behavior. The model depends on simulation-generated data and does not explicitly incorporate physical laws or material constraints, limiting its physical consistency under complex loading and failure conditions.

Hassani Niaki et al. [27] developed a backpropagation DNN to predict the compressive, flexural, and splitting tensile strengths of basalt-fiber and nanoclay-reinforced polymer concrete composites. The study aimed to evaluate the capability of DNNs for mechanical-property prediction using experimentally generated composite data. Three-layer DNN models, along with R^2^, RMSE, MAE, and Partial Dependence Plots (PDPs), were used for prediction and sensitivity analysis. The results demonstrated that DNNs can effectively capture nonlinear relationships and support composite material optimization. The approach was purely data-driven and lacked physics-based constraints, PI loss functions, and explicit failure assessment, limiting its physical consistency and generalization under complex loading conditions. Konale and Srivastava [32] developed a PI Neural Network (PINN) for modeling fracture in elastomers under large deformation. The objective was to overcome mesh dependency and computational complexity associated with conventional FEM-based damage models. The method incorporated governing PDEs, boundary conditions, and constitutive models into PI loss functions using a meshless collocation approach, without requiring training data. The framework effectively modeled crack paths and fracture progression under different defect configurations. This study focuses specifically on elastomer fracture and does not address polymer composites, composite-specific constitutive behavior, or integrated mechanical-response prediction and failure assessment. Gao et al. [23] proposed CINAS-PINN, a PINN that uses causal inference-based neural architecture search to predict fatigue life using welding strain energy. It addresses the fatigue life, and not multi-target mechanical response or failure-index screening, of polymer composites. Jin et al. [11] studied the creep mechanical properties of HTPB solid propellant. Creep is a time-dependent response that the tabular dataset used here does not capture.

### Research Gap

Existing studies have demonstrated the effectiveness of deep learning and physics-informed methods for specific composite constitutive behaviors, multiscale elasticity, fatigue delamination, and damage prediction. However, the reviewed approaches address different material systems and modeling objectives, and the literature reviewed here does not establish a unified framework combining deep feature extraction, physics-informed recurrent prediction, metaheuristic hyperparameter optimization, and failure-criterion-based assessment. The DBN-GJO-PI-RNN framework is designed to integrate these components within a single modeling pipeline. Its contribution is evaluated through comparative predictive metrics and failure assessment, while differences in datasets and loading conditions must be considered when interpreting comparisons with previous studies.

## 3. Proposed Methodology

**Figure 1:**
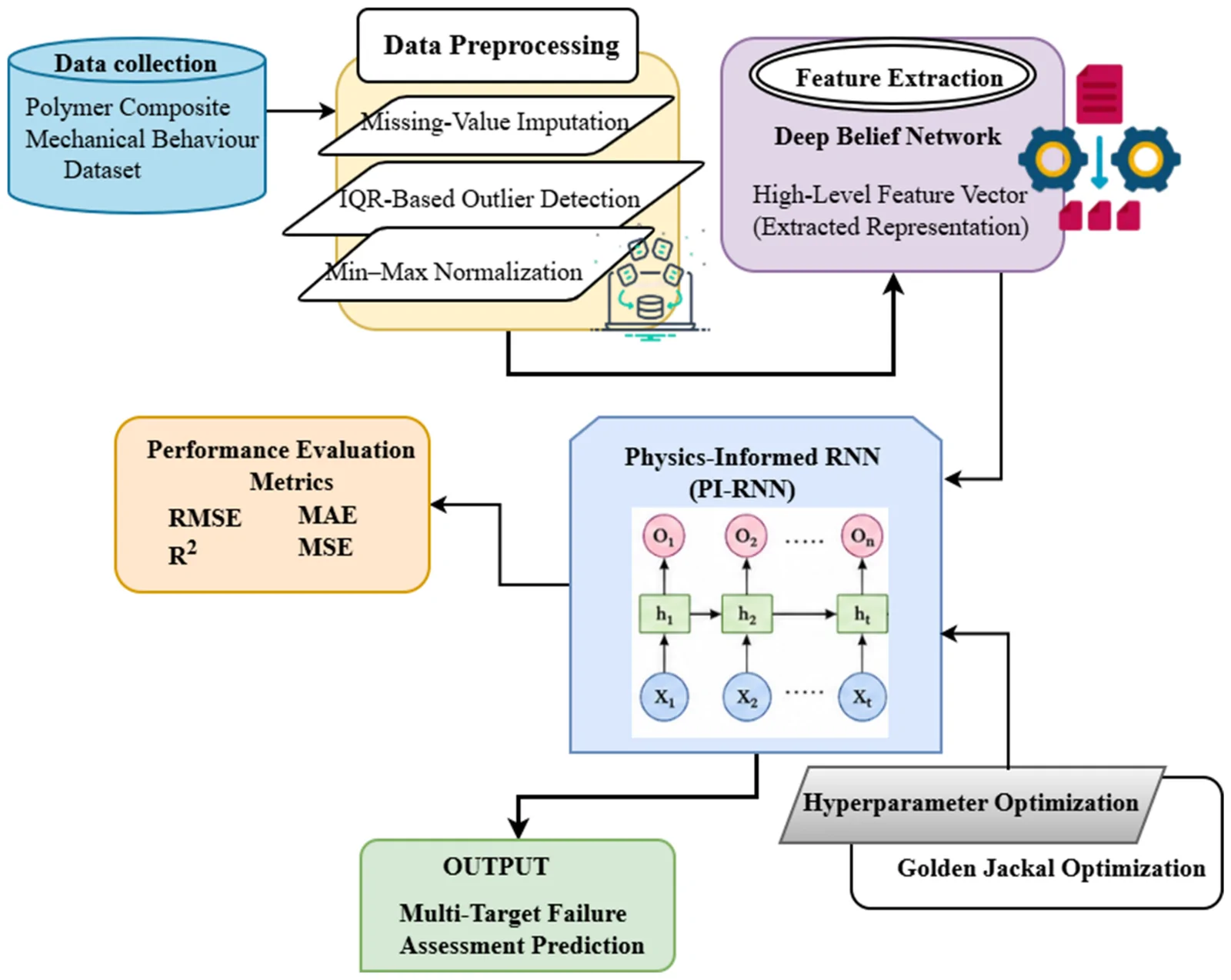
Overall architecture of a developed DBN-GJO-PI-RNN framework.

The overall architecture of the proposed DBN-GJO-PI-RNN framework for polymer-composite mechanical behavior prediction and failure assessment. The workflow comprises data preprocessing, hierarchical feature extraction using a DBN, sequential response modeling through a PI-RNN, and GJO for hyperparameter selection. This optimized framework is evaluated using MAE, MSE, RMSE, and R^2^ and subsequently produces multi-target mechanical-response and failure-assessment predictions, as shown in Figure 1.

### 3.1. DBN-Based Feature Extraction

The preprocessed composite features are transformed into a compact, nonlinear representation using a DBN. The DBN is constructed as a stack of Restricted Boltzmann Machines (RBMs), where each RBM learns progressively higher-level representations of the underlying relationships among material properties, loading conditions, and mechanical responses.

**Figure 2:**
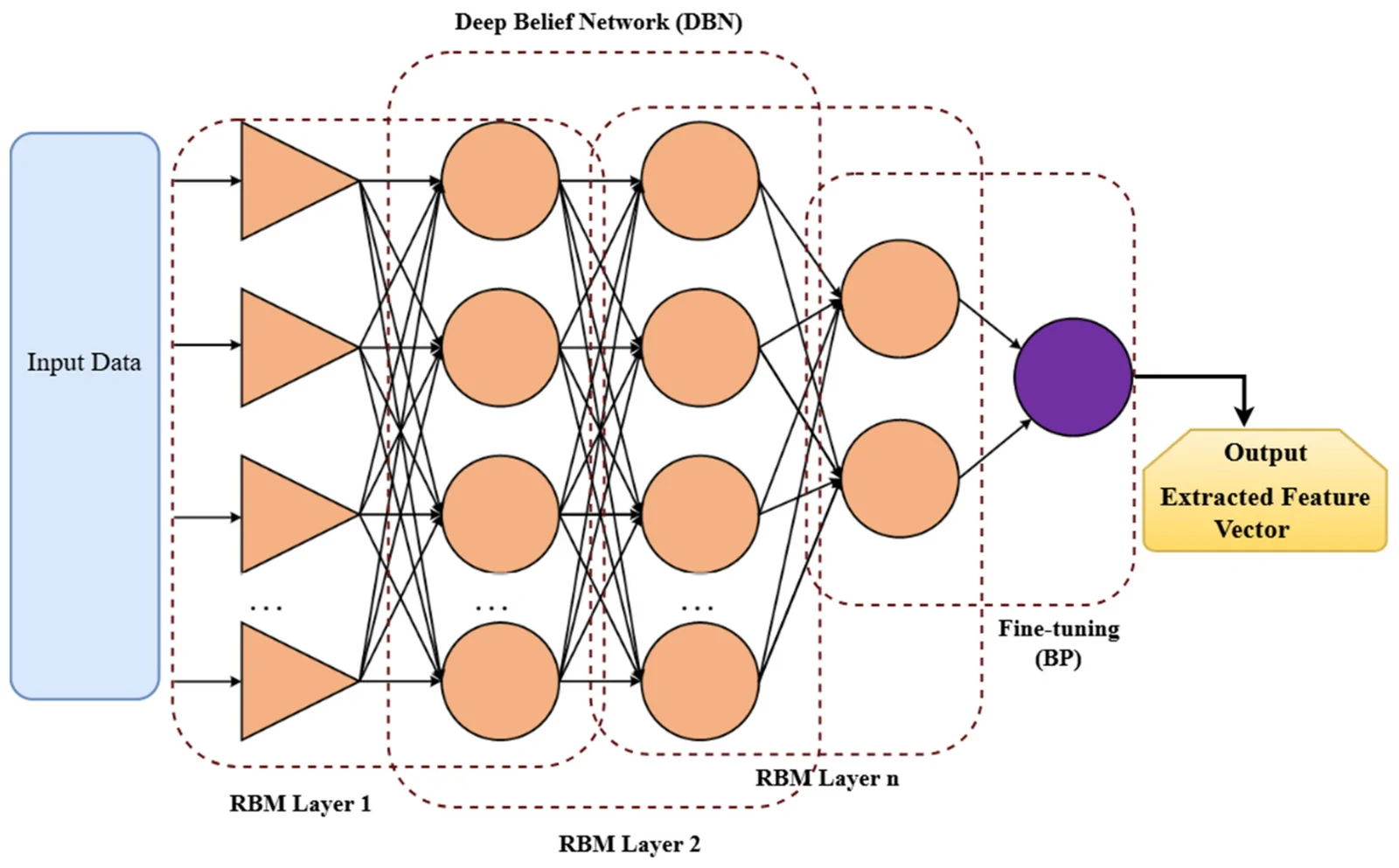
DBN-based hierarchical feature extraction.

Figure 2 illustrates the DBN-based feature extraction process, in which the input polymer-composite data are progressively transformed through stacked Restricted Boltzmann Machine layers. The hierarchical learning process captures nonlinear representations of the material characteristics, and backpropagation fine-tuning further refines the learned parameters to generate a compact high-level feature vector for subsequent prediction.

#### 3.1.1. RBM Energy Function

The energy associated with the visible and hidden units of an RBM is defined as Equation (1):

(1) $$E(v,h)=-\sum_{i=1}^{N_{v}}\;\;\;\sum_{j=1}^{N_{h}}\;\;W_{ij}v_{i}h_{j}-\sum_{i=1}^{N_{v}}\;\;\;a_{i}v_{i}-\sum_{j=1}^{N_{h}}\;\;\;b_{j}h_{j}$$

where E(v,h) is the energy of the RBM state; v is a visible-layer vector; h is a hidden-layer vector; $v_{i}$ is an activation of the i-th visible neuron; $h_{j}$ is the activation of the j-th hidden neuron; $W_{ij}$ is a connection weight between visible neuron i and hidden neuron $j;a_{i}$ is the bias of visible neuron $i_{i}b_{j}$ is the bias of hidden neuron $j;N_{v}$ is the total number of visible neurons; and $N_{h}$ is the total number of hidden neurons.

#### 3.1.2. Hidden-Layer Activation

The probability of activating hidden neuron j given the visible input is calculated as Equation (2):

(2) $$P\left(h_{j}=1|v\right)=\sigma \left(b_{j}+\sum_{i=1}^{N_{v}}\;\;\;W_{ij}v_{i}\right)\;$$

where $P\left(h_{j}=1|v\right)$ is a conditional probability that hidden neuron j is activated, $h_{j}$ is a binary state of hidden neuron $j_{i},\;v$ is a visible-layer input vector, $b_{j}$ is the bias of hidden neuron $j_{i},\;W_{ij}$ is a weight connecting visible neuron i to hidden neuron $j_{i},\;v_{i}$ is the i-th visible input, $N_{v}$ is some visible neurons, and σ(⋅) denotes a sigmoid activation function.

This sigmoid function is expressed as Equation (3):

(3) $$\sigma (z)=\frac{1}{1+e^{-z}}$$

where σ(z) represents the sigmoid output; z denotes a weighted input to a neuron; and e is Euler’s mathematical constant.

#### 3.1.3. Hierarchical Feature Learning

The representations learned by successive DBN layers are obtained using Equation (4):

(4) $$z^{\left(l\right)}=\sigma \left(W^{\left(l\right)}z^{\left(l-1\right)}+b^{\left(l\right)}\right)$$

where $z^{\left(l\right)}$ is a feature vector generated by a l-th hidden layer; $z^{\left(l-1\right)}$ is a feature vector received from the preceding layer; $W^{\left(l\right)}$ is a weight matrix connecting layer l−1 to layer $l;{\;b}^{\left(l\right)}$ is the bias vector of layer $l_{;}l$ denotes a current DBN layer; and σ(⋅) is a sigmoid activation function.

#### 3.1.4. Final Deep Feature Representation

The output of a final DBN layer is used as a high-level representation in Equation (5):

(5) $$F_{DBN}=z^{\left(L\right)}$$

where $F_{DBN}$ denotes a final DBN-derived feature vector; $z^{\left(L\right)}$ represents a feature vector generated by a final hidden layer; and L denotes the overall number of hidden layers in the DBN.

### 3.2. Physics-Guided Constraint Formulation

The DBN-derived features are integrated with the governing physical relationships of polymer composites to ensure that the subsequent PI-RNN predictions remain consistent with established mechanical principles. The physics-guided formulation incorporates constitutive behavior, mechanical equilibrium, and material constraints into the learning process. The physical constraints are incorporated into the learning objective through the constitutive, equilibrium, and boundary-condition residuals defined in Equations (6)–(12). The constitutive residual represents the discrepancy between the predicted mechanical response and the response obtained from the governing material relationship. The equilibrium residual measures the deviation from force balance, while the boundary-condition residual represents the mismatch between predicted and prescribed boundary values. These residuals are combined with the data-fitting loss to guide model training toward predictions that satisfy both the available data and the specified physical constraints. Physical consistency is assessed by examining the magnitude of the individual residuals, with smaller residual values indicating closer agreement with the corresponding governing equations. The dataset contains tabular mechanical-response variables but lacks full-field stress, strain, displacement, and boundary-condition data. Consequently, the complete constitutive stiffness matrix, equilibrium equations, and boundary conditions cannot be independently evaluated. Physics-guided constraints must therefore be limited to calculations supported by available variables and material relationships. Predictive accuracy does not establish physical validity; independent validation requires experimental measurements or verified finite-element simulation data.

#### 3.2.1. Constitutive Relationship

The stress–strain relationship of the composite material can be expressed in general form as Equation (6):

(6) $$\sigma^{phys}=C(\varepsilon ,m)\varepsilon$$

where $\sigma^{phys}$ denotes the physically derived stress vector; C represents the constitutive stiffness matrix of the polymer composite; ε denotes the strain vector; and m represents the material-property vector, including relevant composite material characteristics.

The constitutive residual is then defined as Equation (7):

(7) $$R_{const}=\sigma_{pred}-\sigma^{phys}$$

where $R_{const}$ denotes the constitutive residual; $\sigma_{pred}$ is the stress predicted by the PI-RNN; and $\sigma^{phis}$ is the stress obtained from the constitutive relationship.

#### 3.2.2. Mechanical Equilibrium Constraint

Mechanical equilibrium is imposed through Equation (8):

(8) ∇⋅σ+f=0

where ∇⋅σ denotes the divergence of the stress tensor; σ represents the stress tensor; f denotes the external body-force vector; and 0 represents the equilibrium condition in the absence of unbalanced forces.

The corresponding equilibrium residual is Equation (9):

(9) $$R_{eq}=\nabla \cdot \sigma_{pred}+f$$

where $R_{eq}$ denotes the equilibrium residual; $\sigma_{pred}$ is the predicted stress tensor; ∇⋅(⋅) represents the divergence operator; and f is the applied body-force vector.

#### 3.2.3. Boundary and Material Constraints

The boundary condition is represented as Equation (10):

(10) $$\sigma_{pred}n=t\;on\;\Gamma_{t}$$

where $\sigma_{pred}$ denotes the predicted stress tensor; n is the outward unit normal vector to the boundary; t represents a prescribed traction vector; and $\Gamma_{t}$ denotes a portion of the material boundary where traction is prescribed.

For prescribed displacement conditions in Equation (11):

(11) $$u_{pred}=u_{bc}\;on\;\Gamma_{u}$$

where $u_{pred}$ denotes the predicted displacement vector; $u_{lc}$, represents the prescribed boundary displacement; and $\Gamma_{u}$ denotes the boundary region where displacement is specified.

#### 3.2.4. Overall Physics Residual

The different physical constraints are combined into a unified residual in Equation (12):

(12) $$R_{phys}=R_{const}+R_{eq}+R_{bc}$$

where $R_{phys}$ denotes the overall physics residual; $R_{const}$ represents the constitutive residual; $R_{eq}$ represents the equilibrium residual; and $R_{bc}$ represents the boundary-condition residual.

### 3.3. PI-RNN Development

The extracted DBN features are provided to a PI-RNN to model the sequential and nonlinear evolution of the mechanical response of high-performance polymer composites. This network learns the relationship between material characteristics, loading conditions, and a corresponding stress–strain response while incorporating the physics residuals obtained from the previous stage. The PI-RNN processes input features to predict corresponding mechanical responses; however, the tabular dataset does not establish explicit time steps, loading sequences, or strain histories. Therefore, genuine time-dependent stress–strain learning cannot be claimed without verified sequential data and a clearly defined input order. Creep studies, such as the one on HTPB solid propellant [11], rely on measured time histories of this kind. Without temporal data, the model should be described as predicting mechanical responses rather than validated temporal behavior.

**Figure 3:**
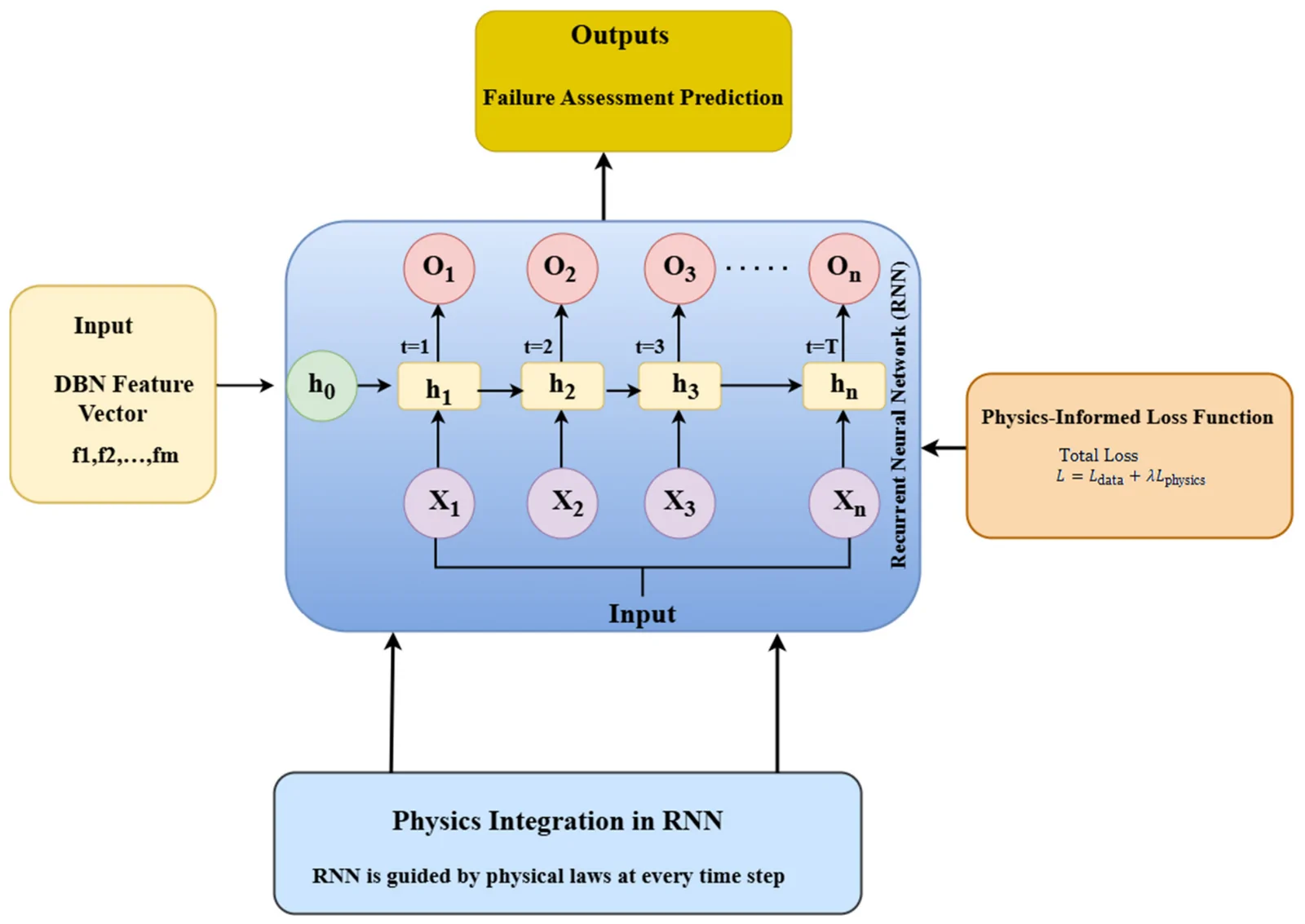
PI-RNN for multi-target prediction.

Figure 3 presents the PI-RNN architecture used for sequential prediction of the composite mechanical responses. The extracted DBN feature vector is provided to the RNN, which processes the input across successive time steps while incorporating physical laws through the PI loss function. The resulting outputs represent the predicted mechanical responses and support subsequent failure assessment.

#### 3.3.1. Recurrent State Representation

The hidden state of the RNN is calculated as Equation (13):

(13) $$h_{t}=tanh\left(W_{xh}x_{t}+W_{hh}h_{t-1}+b_{h}\right)$$

where $h_{t}$ denotes a hidden-state vector at time or loading step $t_{i}x_{t}$ represents an input feature vector at step $t;W_{xh}$ is a weight matrix connecting the input layer to the hidden layer; $W_{hh}$ is a recurrent weight matrix connecting the previous hidden state to a current hidden state; $h_{t-1}$ denotes a hidden state from the preceding step; $b_{h}$ is a hidden-layer bias vector; and tanh(⋅) is a hyperbolic tangent activation function.

The PI-RNN input is formulated as Equation (14):

(14) $$x_{t}=\left[F_{DBN,t},m_{t},l_{t}\right]$$

where $x_{t}$ denotes a complete input vector at a loading step $t;F_{DBN,t}$ represents a DBN-derived feature vector; $m_{t}$ denotes the material-property vector; and $l_{t}$ represents the loading-condition vector.

#### 3.3.2. Mechanical-Response Prediction

The predicted mechanical response is obtained as Equation (15):

(15) $${yˆ}_{t}=W_{hy}h_{t}+b_{y}$$

where ${yˆ}_{t}$ denotes the predicted mechanical response at loading step $t_{i}$; $W_{hy}$ is the weight matrix connecting a hidden layer to the output layer; $h_{t}$ is a current hidden-state vector; and $b_{y}$ is the output-layer bias vector.

For stress–strain prediction, an output can be represented as Equation (16):

(16) $${\sigma ˆ}_{t}=f_{\theta }\left(F_{DBN,t},m_{t},1_{t}\right)$$

where ${\sigma ˆ}_{t}$ denotes the predicted stress response; $f_{\theta }(\cdot )$ represents the PI-RNN parameterized by $\theta_{i}F_{DBN,t}$ denotes the DBN-derived features; $m_{t}$ represents the material properties; and $l_{t}$ denotes the loading conditions.

#### 3.3.3. Physics-Constrained Prediction

The predicted response is evaluated against the physics residuals in Equation (17):

(17) $$R_{PI,t}=G\left({\sigma ˆ}_{t},\varepsilon_{t},m_{t}\right)$$

where $R_{PI,t}$ denotes the physics residual at loading step $t_{i}G(\cdot )$ represents the governing physical operator; ${\sigma ˆ}_{t}$ is the predicted stress; $\varepsilon_{t}$ denotes the strain vector; and $m_{t}$ represents the material-property vector.

The predicted mechanical response from the PI-RNN is optimized by simultaneously considering the agreement with experimental observations and compliance with the governing physical constraints. The total loss combines data loss, physics loss, and constraint loss, thereby reducing prediction errors while improving physical consistency.

The three loss components are combined as Equation (18):

(18) $$L_{total}=\lambda_{d}L_{data}+\lambda_{p}L_{physics}+\lambda_{c}L_{constraint}$$

where $L_{total}$ denotes the overall training loss; $L_{data}$ represents data-fitting loss; $L_{physics}$ represents the physics-consistency loss; $L_{comstraint}$ represents a boundary and material constraint loss; $\lambda_{d}$ denotes a weighting coefficient for a data loss; $\lambda_{p}$ denotes a weighting coefficient for the physics loss; and $\lambda_{c}$ denotes a weighting coefficient for constraint loss.

The PI-RNN parameters are optimized by minimizing $L_{total}$ in Equation (19):

(19) $$\theta^{*}={arg\;min}_{\theta }L_{total}$$

where $\theta^{*}$ denotes the optimized PI-RNN parameter set; θ represents the trainable network parameters; and arg min identifies the parameter set that produces the minimum total loss.

### 3.4. GJO-Based Hyperparameter Optimization

The GJO algorithm is employed to determine an optimal hyperparameter configuration of the PI-RNN. The optimization process searches for a configuration that minimizes the validation objective while maintaining both prediction accuracy and physical consistency. The DBN-GJO-PI-RNN configuration is described using the specified hyperparameter search ranges, population size, maximum iterations, and training settings. Candidate configurations are evaluated using the defined fitness function, and the best configuration is selected based on validation performance and the specified physics-informed constraints. The final configuration includes two DBN hidden layers with 128 and 64 units, a latent dimension of 64, and a two-layer PI-RNN with 128 hidden units. The dropout rate is 0.2, the learning rate is 0.001, and the batch size is 32. The physics-loss weights are 1.0, 0.5, and 0.1, respectively.

#### 3.4.1. Hyperparameter Search Space

The candidate PI-RNN configuration is represented as Equation (20):

(20) $$H=\left[n_{h},L_{r},\eta ,B,D,\lambda_{d},\lambda_{p},\lambda_{c}\right]$$

where H denotes a PI-RNN hyperparameter vector; $n_{h}$ is the number of hidden neurons; $L_{r}$ is the number of recurrent layers; η is a learning rate; B is a batch size; D is a dropout rate; and $\lambda_{d},{\;\lambda }_{p}$, and $\lambda_{c}$ are the weighting coefficients for the data, physics, and constraint losses, respectively.

#### 3.4.2. GJO Fitness Function

The weight coefficients α, β, and γ are optimized by GJO together with the network hyperparameters to balance prediction accuracy, physical consistency, and constraint satisfaction. Higher weights increase the contribution of their corresponding loss components to the fitness function, influencing the configurations selected during optimization. The optimal coefficients are those that minimize the combined validation objective within the predefined search bounds.

Each candidate hyperparameter configuration is evaluated using a combined validation objective in Equation (21):

(21) $$F(H)=\alpha L_{val}+\beta L_{physics}+\gamma L_{constraint}$$

where F(H) denotes the fitness value of the candidate configuration; $L_{val}$ represents the validation prediction loss; $L_{physics}$ denotes the physics-residual loss; $L_{constraint}$ represents the constraint loss; and $\alpha_{r}$ β, and γ are weighting coefficients controlling the contribution of the three objectives.

The optimal hyperparameter configuration is obtained as Equation (22):

(22) $$H^{*}={arg\;min}_{H\in \Omega }F(H)$$

where $H^{*}$ denotes the optimal PI-RNN hyperparameter configuration; Ω represents the predefined hyperparameter search space; and F(H) is the fitness value associated with a candidate configuration.

#### 3.4.3. GJO Position Update

During optimization, the position of each jackal in the search space is updated according to Equation (23):

(23) $$X_{i}^{t+1}=X_{p}^{t}-E\left|JX_{p}^{t}-X_{i}^{t}\right|$$

where $X_{i}^{t+1}$ denotes the updated position of jackal i at iteration $t+1;X_{i}^{t}$ represents its current position; $X_{p}^{t}$ denotes the position of the selected prey at iteration $t_{i}$: E represents the escaping-energy coefficient controlling exploration and exploitation; J denotes the prey-escape coefficient; and t represents the current optimization iteration.

#### 3.4.4. Optimal Model Selection

After evaluating the candidate configurations over successive iterations, the configuration producing the minimum fitness value is selected in Equation (24):

(24) $$F^{*}=\underset{H\in \Omega }{min}\;F(H)$$

where $F^{*}$ denotes the minimum fitness value obtained during optimization; H represents a candidate hyperparameter configuration; and Ω denotes the hyperparameter search space. The GJO parameters were optimized using a population size search range of 10–50 and a maximum iteration range of 25–150. The optimal configuration was a population size of 30 and 100 iterations, providing the settings used for model optimization and supporting reproducibility. Algorithm 1 determines the optimal PI-RNN configuration by minimizing the validation fitness through iterative parameter updates.
**Algorithm 1:** GJO-Based Hyperparameter Optimization of PI-RNNInput:    Training dataset D_train_    Validation dataset D_val_    Search space H    Population size P    Maximum iterations T Output:    Optimal hyperparameter set H* Initialize P Golden Jackal positions within search space H Evaluate the fitness of each jackal using validation loss for t = 1 to T do  Identify the best jackal position H_best_  Identify the second-best jackal position H_second_ for i = 1 to P do Update the position of jackal i Keep updated parameters within their predefined bounds Construct the PI-RNN using the updated parameters Train the PI-RNN using D_train_ Calculate data, physics, and constraint losses Evaluate the combined fitness using D_val_ end for Update H_best_ according to the minimum fitness value end for Return H* = H_best_

### 3.5. Predictive Modeling and Failure Assessment

The GJO-optimized PI-RNN is used to predict the mechanical response of high-performance polymer composites under unseen material and loading conditions. The predicted stress–strain behavior is subsequently evaluated using an appropriate failure criterion to identify the onset and progression of material failure. The failure assessment uses the failure index (FI), where FI ≥ 1 indicates the onset of the corresponding failure criterion. The framework evaluates failure onset using the predicted mechanical responses; however, the dataset contains only test samples with FI values ranging from 1.14 to 2.25. Therefore, the model’s ability to distinguish safe states (FI < 1) from failed states has not been validated.

#### 3.5.1. Mechanical-Response Prediction

The optimized PI-RNN predicts the mechanical response as Equation (25):

(25) $${yˆ}_{t}=f_{\theta^{*}}\left(F_{DBN,t},m_{t},l_{t}\right)$$

where ${yˆ}_{t}$ denotes the predicted mechanical response at loading step $t_{i}f_{\theta^{*}}(\cdot )$ represents the GJO-optimized PI-RNN; $\theta^{*}$ denotes the optimized trainable parameters; $F_{DBN,t}$ represents the DBN-derived feature vector; $m_{t}$ denotes the material-property vector; and $l_{t}$ represents the loading-condition vector.

#### 3.5.2. Failure-Criterion Evaluation

For composite failure assessment, a suitable failure index can be defined using the predicted stress state. For example, the Hashin failure criterion can be employed to evaluate different failure modes.

For fiber-tension failure in Equation (26):

(26) $$FI_{ft}={\left(\frac{\sigma_{11}}{X_{t}}\right)}^{2}+{\left(\frac{\tau_{12}}{S}\right)}^{2}$$

where $FI_{ft}$ denotes the fiber-tension failure index; $\sigma_{11}$ represents the longitudinal stress along the fiber direction; $X_{t}$ denotes a longitudinal tensile strength; $\tau_{12}$ represents the in-plane shear stress; and S denotes the in-plane shear strength.

For matrix-tension failure in Equation (27):

(27) $$FI_{mt}={\left(\frac{\sigma_{22}}{Y_{t}}\right)}^{2}+{\left(\frac{\tau_{12}}{S}\right)}^{2}$$

where $FI_{mt}$ denotes the matrix-tension failure index; $\sigma_{22}$ represents the transverse stress; $Y_{t}$ denotes the transverse tensile strength; $\tau_{12}$ represents an in-plane shear stress; and S denotes the in-plane shear strength.

Failure initiation is determined by Equation (28):

(28) FI≥1

where FI denotes the applicable failure index. A value below unity indicates that the corresponding failure criterion has not been reached, whereas a value equal to or greater than unity indicates the onset of the corresponding failure mode. Algorithm 2 uses the optimized configuration to predict the six mechanical-response targets and subsequently performs failure assessment based on the predicted responses.
**Algorithm 2:** DBN-GJO-PI-RNN Multi-Target PredictionInput:    Preprocessed dataset D    Six target variables Y = {y1, y2, …, y6}    Optimal hyperparameters H* Output:    Predicted responses and evaluation metrics 1: Train the DBN using the input features 2: Extract high-level latent representations Z 3: for j = 1 to 6 do 4:     Select target variable y_j_ 5:     Combine Z with the corresponding physical and loading information 6:     Initialize the PI-RNN using H∗ 7:     Train the PI-RNN using the data and PI constraints 8:     Generate predictions $\hat{y}$_j_ for target y_j_ 9:     Calculate MAE, MSE, RMSE, and R^2^ 10:    Store predictions and evaluation metrics for y_j_ 11: end for 12: Aggregate the target-wise prediction results 13: Perform failure assessment using the predicted mechanical responses 14: Return all predictions, evaluation metrics, and failure-assessment results

## 4. Experimental Setup

### 4.1. Dataset Description

The Polymer-Composite Mechanical Behavior Dataset [34], obtained from Kaggle, is used in this study to model the mechanical behavior of polymer composites using the proposed DBN-GJO-PI-RNN framework. The dataset contains 5000 samples and 15 columns, providing diverse material and mechanical-response information suitable for learning nonlinear relationships between composite characteristics and mechanical behavior. The dataset is appropriate for the proposed methodology because its structured numerical attributes support DBN-based feature extraction, PI sequential modeling, and GJO-based hyperparameter optimization. The availability of multiple mechanical-response variables also enables a comprehensive evaluation of prediction accuracy and subsequent failure assessment. The dataset comprises 5000 samples, including nine input features and six output targets. Column definitions, units, and data provenance should be documented using the original dataset source. Missing values and outliers must be handled using verified preprocessing procedures, with imputation, scaling, and thresholds determined from training data only to prevent information leakage.

Table 1 summarizes the dataset characteristics, data partitioning, and preprocessing procedures used in the proposed framework. The dataset contains 5000 samples and 15 columns, including six response targets, obtained from Kaggle. The data are divided into training, validation, and testing sets in a 70:15:15 ratio. Missing-value handling, interquartile range (IQR)-based outlier analysis, and Min–Max scaling are applied to improve data quality and ensure consistent feature ranges.

Table 2 summarizes the dataset size, input features, output variables, and parameters used for Hashin-based failure assessment. It lists the fiber, matrix, and shear strength values, together with the failure-index threshold and the reported test range.

### 4.2. Data Preprocessing

The raw polymer-composite dataset is first processed to remove inconsistencies, handle missing observations, identify abnormal samples, and transform the numerical features into a common scale. This preprocessing improves data quality and prevents features with larger numerical ranges from dominating the subsequent DBN and PI-RNN models.

#### 4.2.1. Missing-Value Imputation

Missing observations are replaced using the mean of the available observations for the corresponding feature in Equation (29):

(29) $${xˆ}_{ij}=\frac{1}{N_{j}}\sum_{\begin{array}{c} k=1\\ x_{kj}\neq NA \end{array}}^{N}\;\;\;x_{kj}$$

where ${xˆ}_{ij}$ denotes an imputed value for i-th sample and j-th feature; $x_{kj}$ represents an observed value of feature j for sample $k_{i}N_{j}$ is the number of non-missing observations for feature j;N denotes the total number of samples; and NA indicates a missing observation.

#### 4.2.2. Interquartile Range (Iqr)-Based Outlier Detection

Potential outliers are identified using an IQR criterion in Equation (30):

(30) $$IQR_{j}=Q_{3,j}-Q_{1,j}$$

where $IQR_{j}$ denotes the interquartile range of feature $j;Q_{1,j}$ and $Q_{3,j}$ represent the first and third quartiles of feature $j_{i}$ respectively.

#### 4.2.3. Min–Max Feature Normalization

After outlier treatment, the numerical features are normalized to the interval [0, 1] using Equation (31):

(31) $$x_{ij}^{norm}=\frac{x_{ij}-x_{j}^{min}}{x_{j}^{max}-x_{j}^{min}}$$

where $x_{ij}^{norm}$ denotes a normalized value of feature j for sample $i_{i}x_{ij}$ represents an original feature value; $x_{j}^{min}$ and $x_{j}^{max}$ denote the minimum and maximum values of feature j, respectively. This transformation places all input features on a comparable scale and facilitates stable learning by the subsequent DBN feature-extraction model.

### 4.3. Computational Environment

The proposed DBN-GJO-PI-RNN algorithm has been implemented using a desktop computer system that contains an Intel^®^ Core™ i9-14900K processor working at 3.20 GHz, with 24 cores, including 8 Performance cores and 16 Efficiency cores, 16 GB RAM, and a 64-bit x64-based architecture. The experiments have been performed using Windows 10 (10.0.26200), where the computations are done using the CPU and no GPU acceleration has been used. The software environment includes Python 3.12.12, PyTorch 2.11.0+cpu, NumPy 2.0.2, Pandas 2.2.2, Scikit-learn 1.6.1, and Matplotlib 3.10.0 with a compatible SciPy library.

### 4.4. Hyperparameter Configuration

The hyperparameters and optimal configuration adopted for the proposed DBN-GJO-PI-RNN framework. Min–Max normalization and a 70:15:15 data split is used for consistent model development, and the evaluation is shown in Table 3.

The DBN employs two hidden layers with 128 and 64 units and a 64-dimensional latent representation to extract hierarchical composite features. The PI-RNN uses two recurrent layers with 128 hidden units and a dropout rate of 0.20, with a learning rate of 0.001. The PI loss assigns weights of 1.0,0.5, and 0.1 to the data, physics, and constraint terms, respectively, enabling balanced consideration of prediction accuracy and physical consistency. For GJO, population sizes of 30 and 100 iterations are employed, while the optimization considers the selected architectural, learning-rate, dropout, and loss-weight parameters. The combined prediction error and physics residual are used as the optimization objective, allowing GJO to identify a suitable configuration for improving the predictive and physics-consistent performance of the proposed framework.

### 4.5. Performance Evaluation Metrics

The proposed DBN-GJO-PI-RNN framework is evaluated by comparing its predicted mechanical responses with the corresponding experimental or validated reference responses. The evaluation considers both predictive accuracy and physics consistency to verify the success and reliability of the proposed framework.

This prediction error is first quantified using MAE in Equation (32):

(32) $$MAE=\frac{1}{N}\sum_{i=1}^{N}\;\;\;\left|y_{i}-{yˆ}_{i}\right|\;$$

where MAE denotes the MAE; N represents the total number of test samples; $y_{i}$ denotes the experimentally observed mechanical response for sample $i_{i}$; and ${yˆ}_{i}$ represents a corresponding response predicted by a developed model.

MSE is expressed by Equation (33),

(33) $$MSE=\frac{1}{N}\sum_{i=1}^{N}\;\;{\left(y_{i}-{yˆ}_{i}\right)}^{2}$$

where N denotes the number of observations, $y_{i}$ represents an actual response, and ${yˆ}_{i}$ denotes a predicted response. Lower MSE indicates smaller prediction errors.

The RMSE is calculated as Equation (34):

(34) $$RMSE=\sqrt{\frac{1}{N}\;\;{\sum_{i=1}^{N}\;\left(y_{i}-{yˆ}_{i}\right)}^{2}}$$

where RMSE denotes the RMSE; N is a number of test samples; $y_{i}$ is an observed response; and ${yˆ}_{i}$ is a predicted response. Lower RMSE indicates smaller prediction errors.

The coefficient of determination $R^{2}$ is defined as Equation (35):

(35) $$R^{2}=1-\frac{\sum_{i=1}^{N}\;\;\;{\left(y_{i}-{yˆ}_{i}\right)}^{2}}{\sum_{i=1}^{N}\;\;\;{\left(y_{i}-y^{¯}\right)}^{2}}$$

where $R^{2}$ denotes the coefficient of determination; $y_{i}$ represents the observed response; ${yˆ}_{i}$ denotes the predicted response; $y^{¯}$ represents the mean of the observed responses; and N denotes the number of test samples. A higher $R^{2}$ indicates better agreement between predicted and observed responses.

## 5. Results and Discussion

### 5.1. Model Convergence and Optimization Analysis

The decreasing and stabilizing loss values confirm stable convergence and effective optimization of the proposed framework.

**Figure 4:**
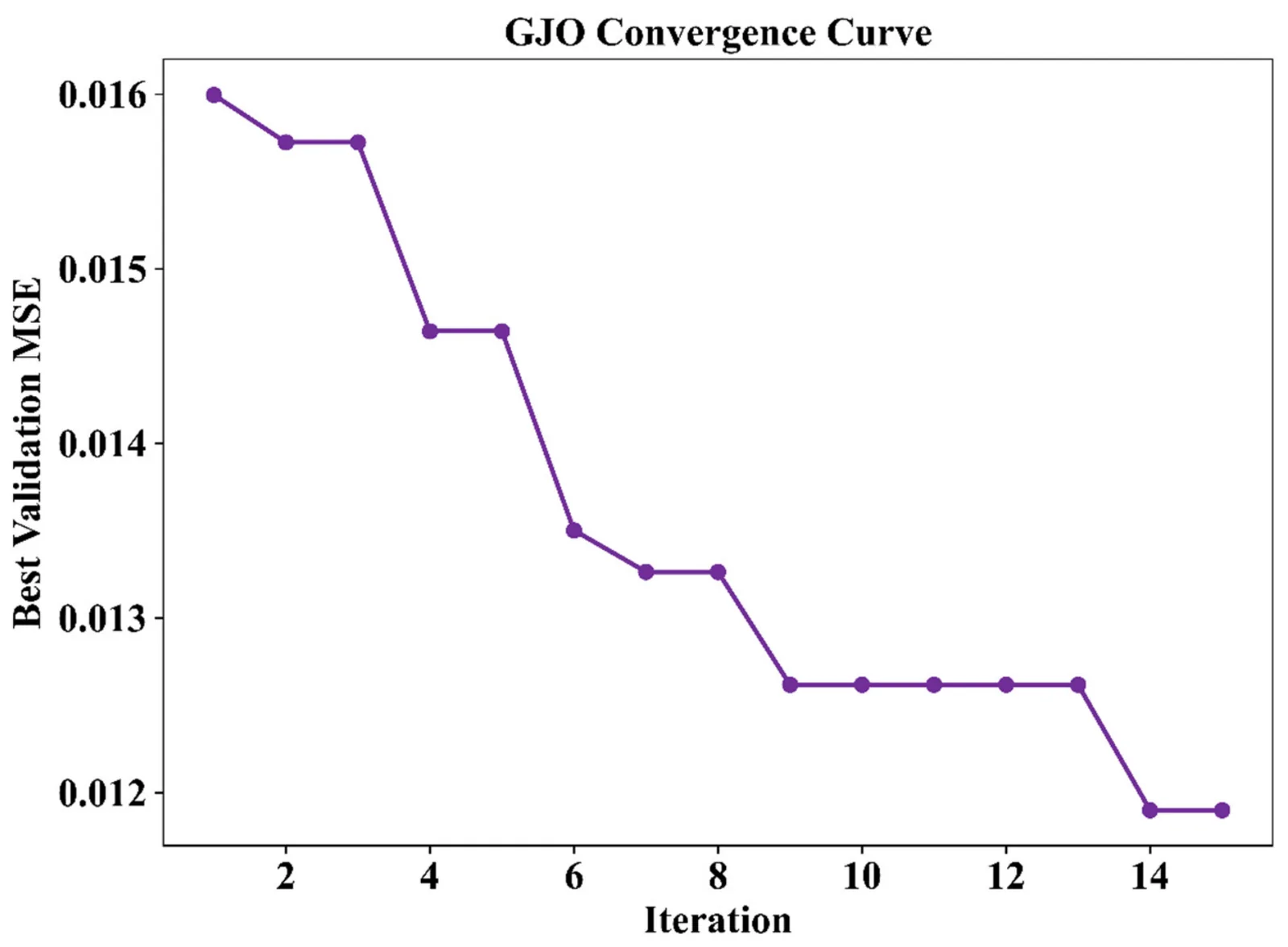
GJO convergence curve for PI-RNN hyperparameter optimization.

Figure 4 illustrates the convergence of the Golden Jackal Optimization (GJO) algorithm during PI-RNN hyperparameter optimization. The validation MSE decreases from 0.0160 in the first iteration to 0.0119 by iterations 14–15, indicating progressive improvement in the selected configuration. The subsequent stabilization suggests that the search has reached a consistent solution within the specified optimization range. However, convergence alone does not establish a global optimum or robustness across different initializations.

**Figure 5:**
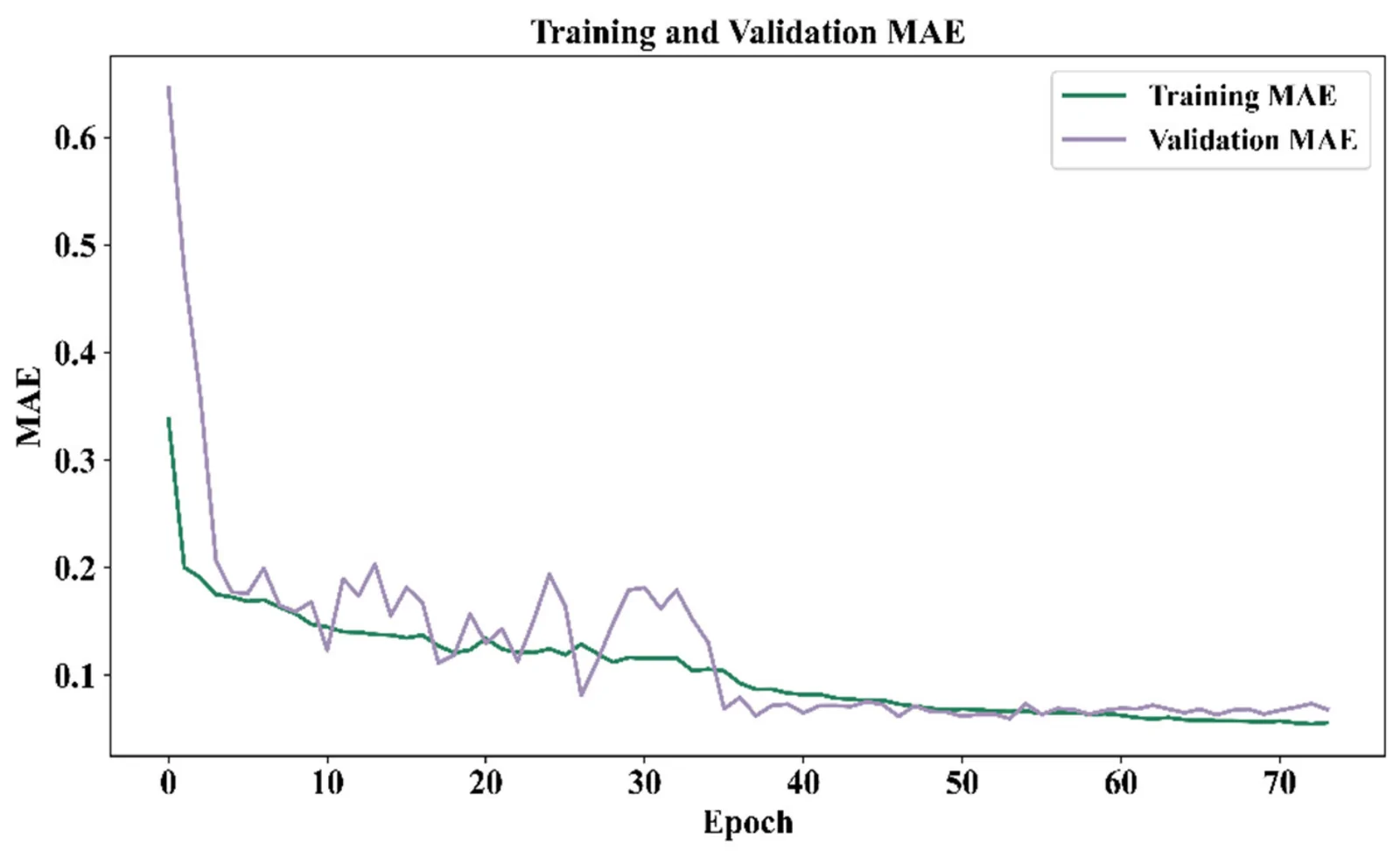
Training and validation MAE convergence during model training.

Figure 5 shows that the training MAE decreases from approximately 0.34 to 0.05–0.06, while the validation MAE falls from approximately 0.65 to 0.05–0.07. Both curves stabilize after around 40 epochs, indicating that the model progressively reduces prediction errors and achieves a stable training process. The relatively close final values suggest limited divergence between training and validation performance. The small fluctuations near convergence also indicate that minor variations in prediction error remain.

**Figure 6:**
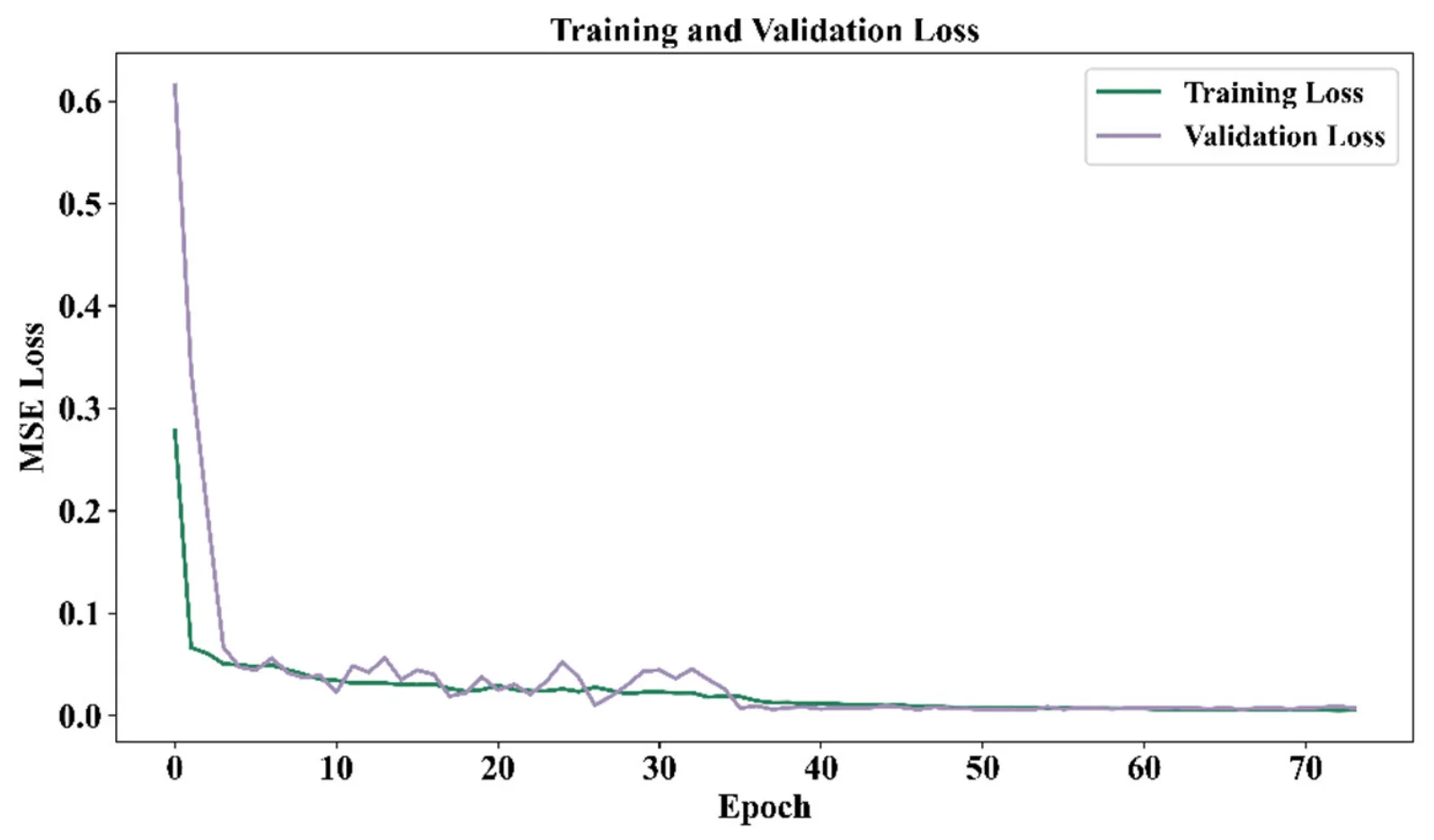
Training and validation MSE loss convergence.

Figure 6 shows the training and validation MSE convergence of the proposed PI-RNN. The training MSE decreased from 0.28 to approximately 0.01, while the validation MSE decreased from 0.61 to approximately 0.00–0.01, with both curves stabilizing after around 40 epochs. These convergence losses were calculated using normalized target values, whereas the reported test MSE of 1.9494 was calculated after inverse transformation to the original target scales; therefore, the values are not directly comparable. The similar convergence trends indicate stable optimization, although independent test results are needed to confirm generalization and assess potential overfitting.

**Figure 7:**
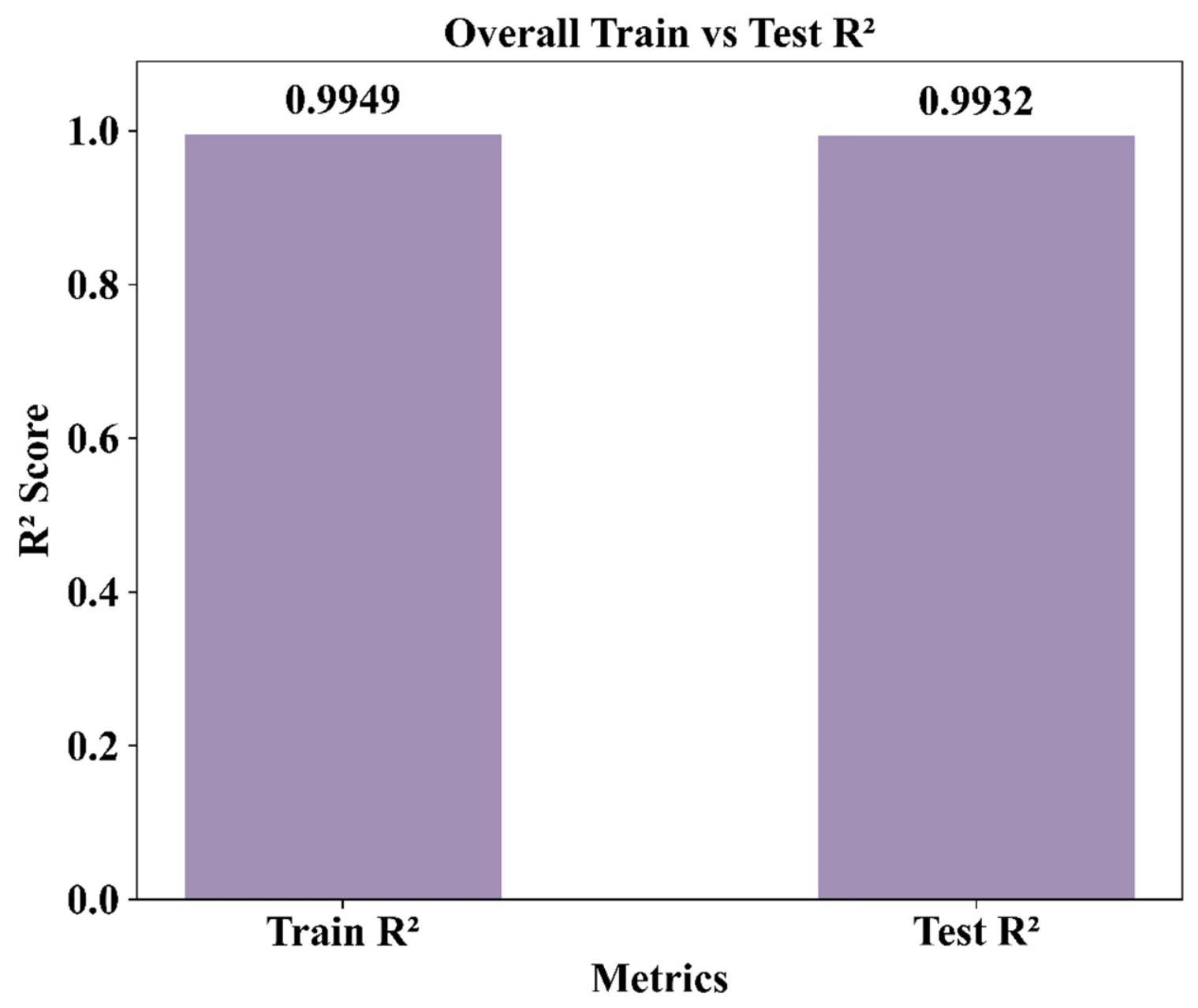
Overall training and testing R^2^ performance.

Figure 7 compares the overall coefficient of determination R^2^ for the training and testing datasets. The model achieves (R^2^ = 0.9949) during training and (R^2^ = 0.9932) during testing, giving a difference of only 0.0017. This close agreement indicates that the model retains a high level of predictive accuracy on the held-out test data, with limited performance degradation relative to training. However, these results establish generalization only for the evaluated data partition; additional cross-validation and testing on independent datasets containing different material and loading conditions are needed to establish broader applicability.

**Figure 8:**
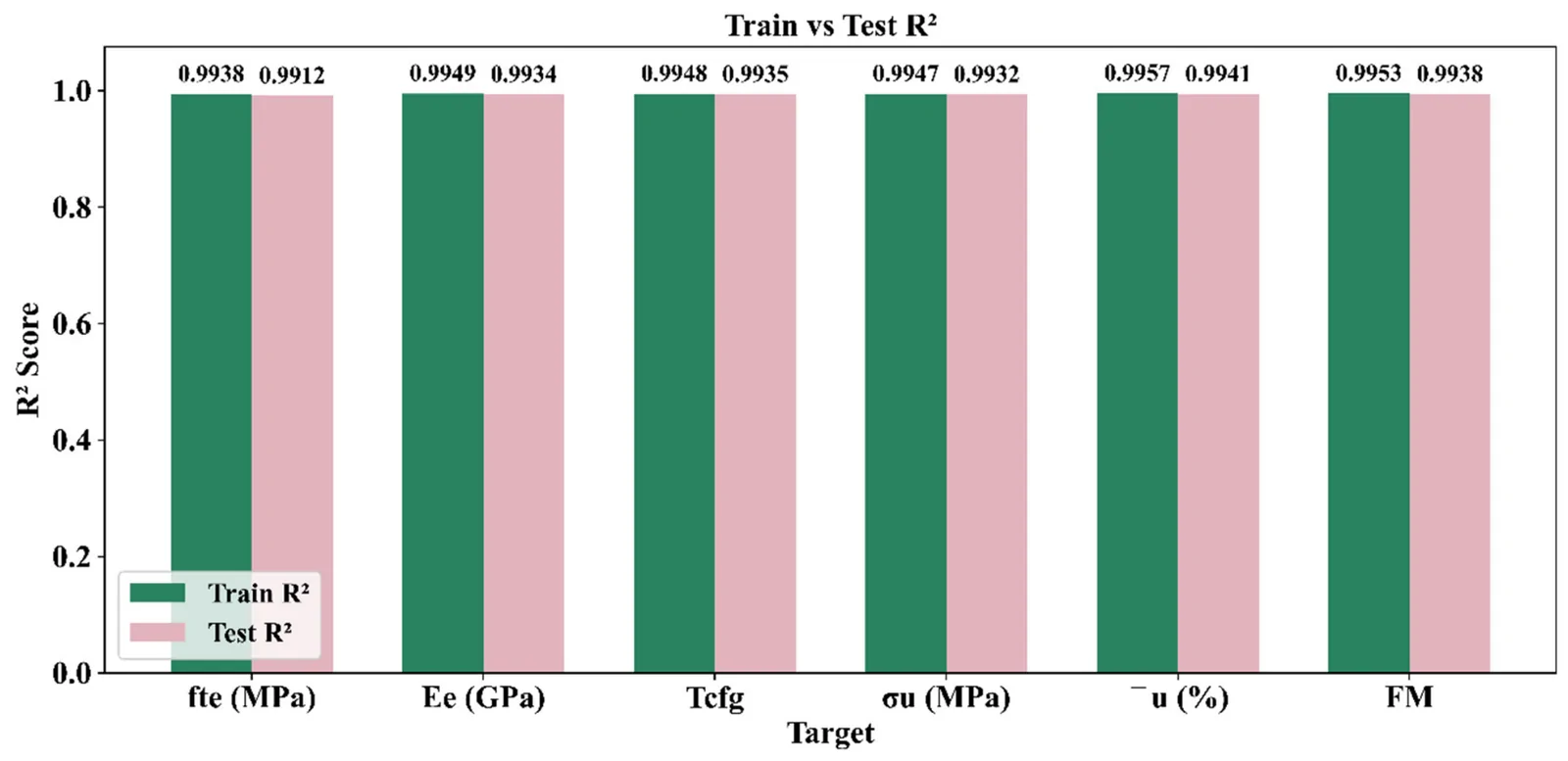
Target-wise comparison of training and testing $R^{2}$ scores.

The target-wise $R^{2}$ analysis demonstrates consistently high predictive performance across all six mechanical-property targets. For $f_{te}(MPa)$, the training and testing $R^{2}$ values are 0.9938 and 0.9912, respectively, as illustrated in Figure 8. For $E_{e}(GPa)$, the corresponding values are 0.9949 and 0.9934, while $T_{efg}$ achieves 0.9948 and 0.9935. The $\sigma_{u}(MPa)$ target obtains 0.9947 and 0.9932, whereas $\varepsilon_{u}(\%)$ achieves 0.9957 and 0.9941. Finally, FM obtains training and testing $R^{2}$ values of 0.9953 and 0.9938, respectively. These consistently small differences between training and testing scores indicate stable learning and strong generalization across the different composite-response targets.

The predictive performance of the DBN-GJO-PI-RNN framework is evaluated separately for all six mechanical-response targets using mean absolute error (MAE), mean squared error (MSE), root mean squared error (RMSE), and the coefficient of determination ($R^{2}$). The reported target-wise RMSE values are 0.6780 for fte (MPa), 0.0367 for Ee (GPa), 0.0087 for Tefg, 7.5989 for u (MPa), 0.0394 for u (%), and 0.0154 for FM. These results show that prediction errors vary across targets.

The predicted and reference values should be converted back to their original physical units before calculating target-wise errors, where inverse transformation is part of the implemented evaluation procedure. Because the targets have different units and numerical scales, their error magnitudes should be interpreted individually rather than compared directly. The overall MAE, MSE, RMSE, and ($R^{2}$) are reported as 1.1132, 1.9494, 1.3962, and 0.9932, respectively. The method used to aggregate target-wise errors must be stated explicitly, and the overall metrics should be interpreted in accordance with that calculation method.

The DBN-GJO-PI-RNN framework was evaluated using a 70:15:15 training, validation, and testing split, comprising 3500, 750, and 750 samples, respectively. The overall training and testing ($R^{2}$) values were 0.9949 and 0.9932, respectively, with a difference of 0.0017. The test-set MAE, MSE, and RMSE were 1.1132, 1.9494, and 1.3962, respectively. These results demonstrate predictive agreement on the selected test split. However, evaluation using a single data partition does not establish robustness across independent splits or external datasets.

### 5.2. Predictive Performance and Failure Assessment

The proposed framework evaluates six mechanical-response targets using standard regression metrics and assesses the corresponding failure behavior using the predicted responses.

**Figure 9:**
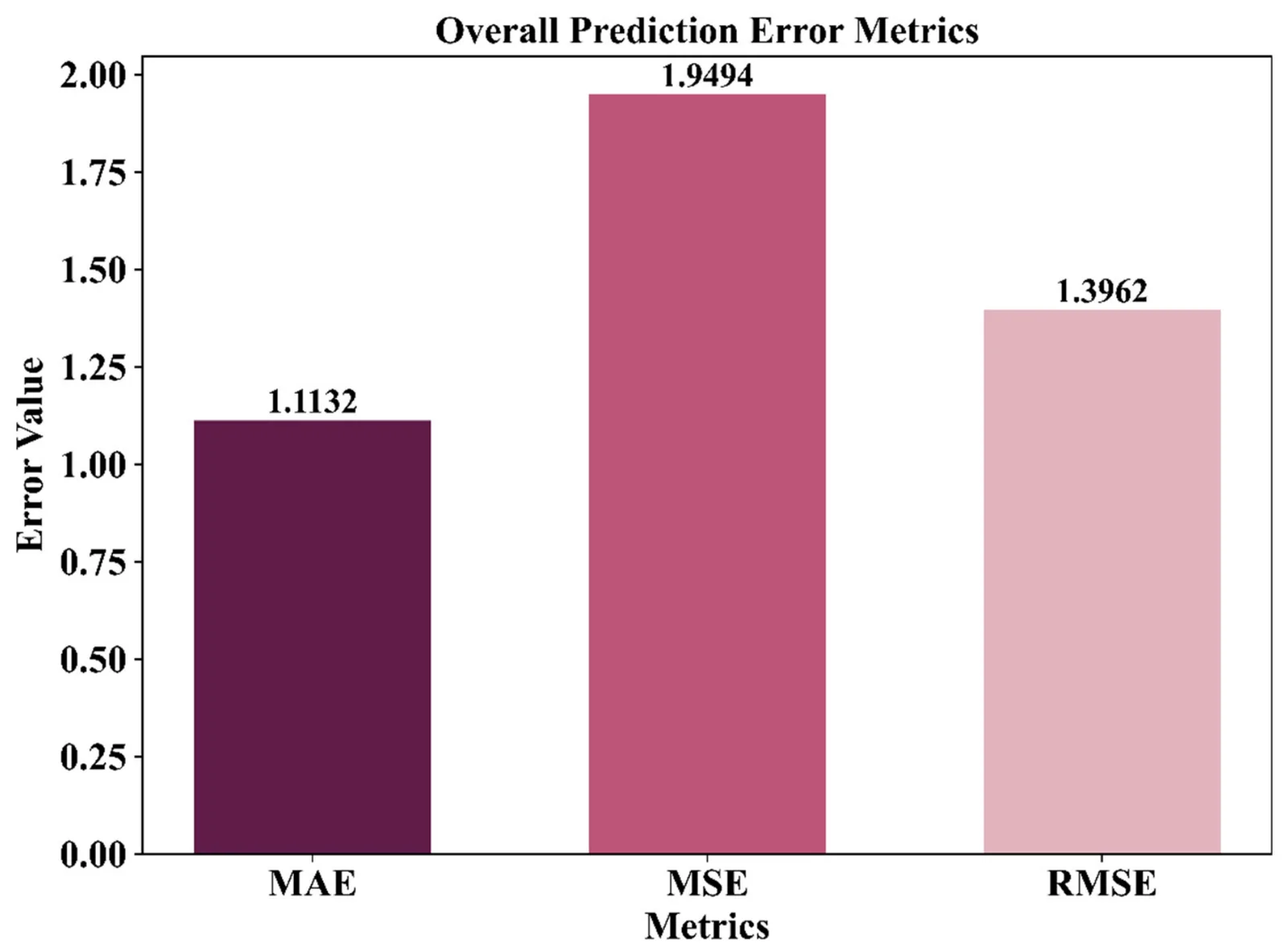
Overall prediction error metrics of the proposed DBN-GJO-PI-RNN framework.

The prediction performance of the proposed DBN-GJO-PI-RNN framework is illustrated in Figure 9 using MAE, MSE, and RMSE across the six mechanical response variables. The proposed model achieved an overall MAE of 1.1132, MSE of 1.9494, and RMSE of 1.3962. These overall metrics were calculated from the pooled prediction errors of all six target variables after inverse transformation to their original physical units; therefore, the overall RMSE represents the aggregate multi-target prediction error and is not the RMSE of any individual target. The results demonstrate a framework for multi-target mechanical-response prediction.

**Figure 10:**
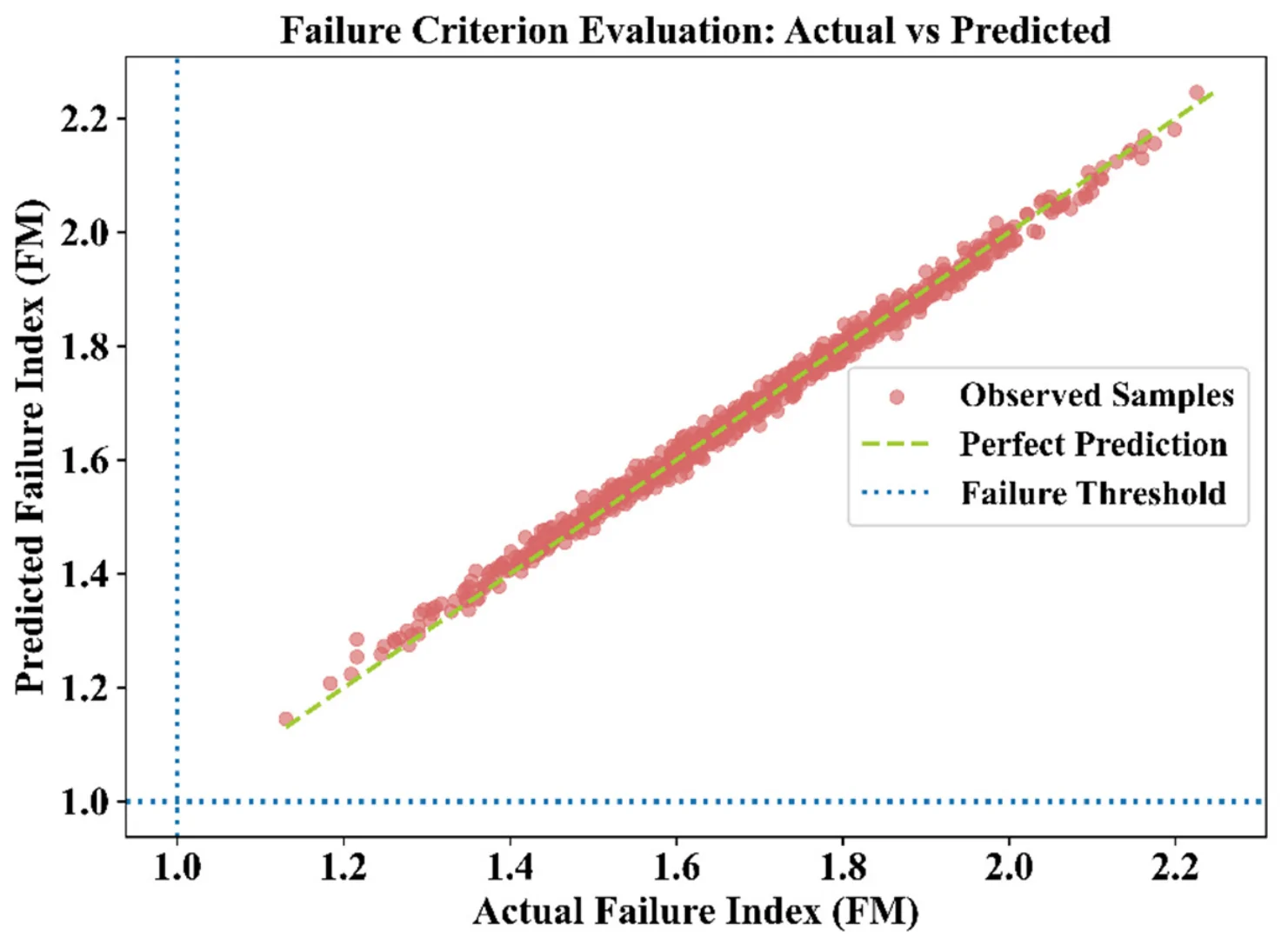
Failure index prediction and assessment.

The failure prediction performance of the proposed DBN-GJO-PI-RNN model for Failure Index (FM) prediction is shown in Figure 10. The true values of FM vary from 1.14 to 2.25, while the predicted ones lie close to the line of perfect prediction. All examples lie above the failure threshold, which equals 1.0 for the FM indicator.

**Figure 11:**
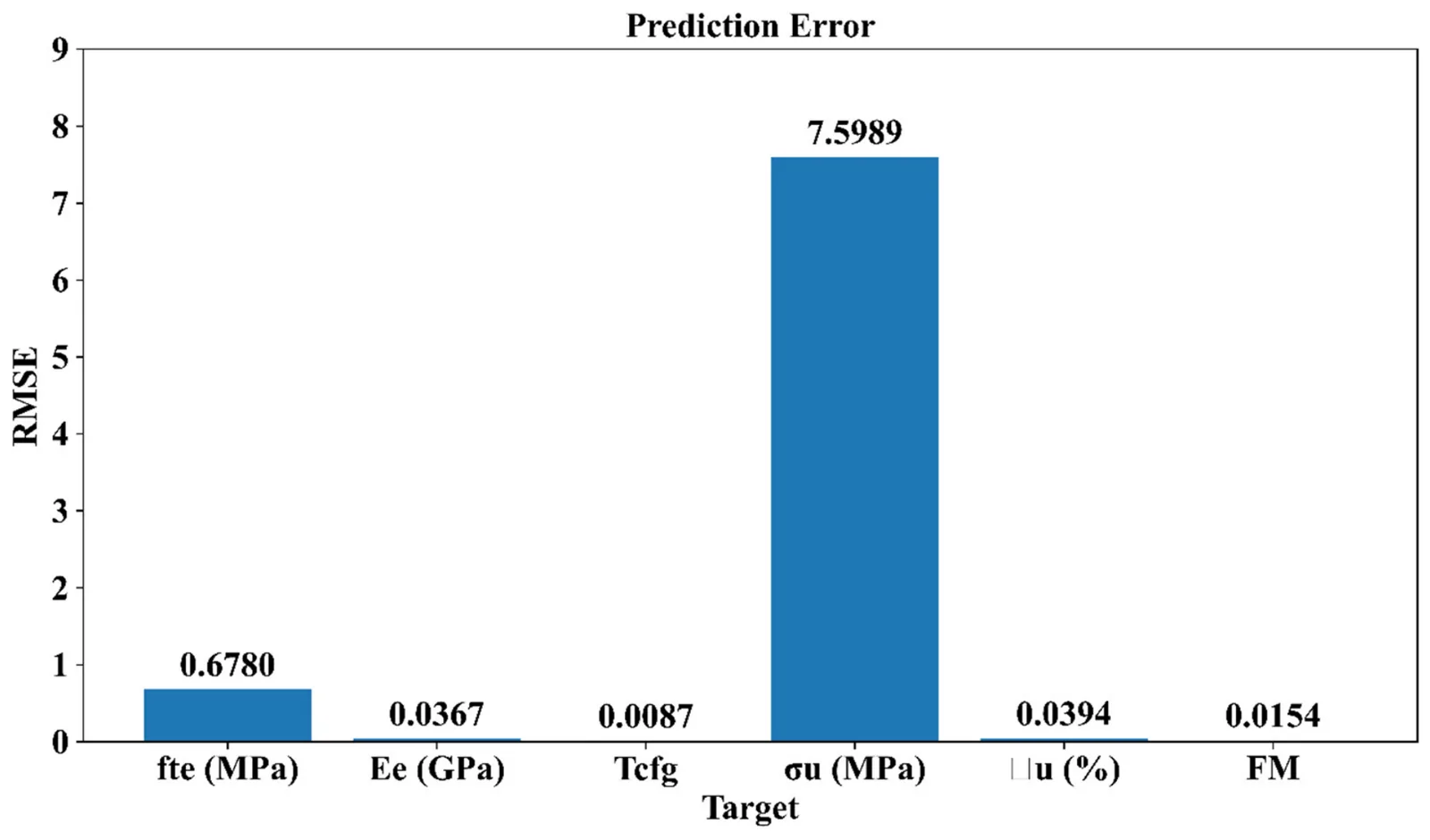
Target-wise RMSE analysis of the proposed framework.

The target-wise RMSE results of Figure 11 indicate that the proposed framework provides different levels of prediction error across the considered mechanical properties. The RMSE values are 0.6780 for $f_{te}(MPa{)}_{t}$, 0.0367 for $E_{e}$ (GPa), 0.0087 for $T_{efg},7.5989$ for $\sigma_{u}(MPa{)}_{r},\;$0.0394 for $\varepsilon_{u}(\%)$, and 0.0154 for FM. The lowest prediction error is observed for $T_{efg}$ with a RMSE of 0.0087, followed by FM (0.0154) and $E_{e}$(0.0367). The largest absolute RMSE is observed for $\sigma_{u}$ at 7.5989 MPa, which may be associated with the larger numerical scale and variability of this target. These results demonstrate that the proposed framework maintains low prediction errors across the different mechanical-response variables.

These comparative results demonstrate a superior performance of the developed DBN-GJO-PI-RNN over the evaluated baseline models as shown in Table 4. This proposed model achieves the lowest MAE (1.1132), MSE (1.9494), and RMSE (1.3962), together with the highest R^2^ (0.9932). In comparison, 1D-CNN, BiLSTM, GRU, TCN, PI-RNN, and DBN-PI-RNN obtain progressively lower predictive performance. The improvement indicates that DBN-based feature extraction, PI sequential modeling, and GJO-based optimization effectively enhance prediction accuracy and generalization for high-performance polymer composites. All baseline models and ablation variants were evaluated using identical training, validation, and testing data partitions. The same preprocessing procedures, optimization budgets, and evaluation metrics were maintained to ensure a fair comparison. This consistent evaluation setup enables reliable comparison of the proposed model against the baseline models.

Table 5 summarizes the optimization settings and loss-function weights used in the DBN-GJO-PI-RNN framework. The Golden Jackal Optimization algorithm operates with a population of 30 and a maximum of 100 iterations. The loss function assigns weights of 1, 0.5, and 0.1 to the data, physics, and constraint components, respectively.

Table 6 presents the search ranges and selected configurations of the DBN-GJO-PI-RNN framework. The DBN and PI-RNN architectures are configured by selecting the number of layers, hidden units, and latent dimension. Training parameters include learning rate, batch size, dropout rate, and maximum epochs. The selected values define the final model configuration used for training and prediction.

Table 7 compares training time, hardware requirements, computational complexity, and predictive performance across conventional and proposed models. The proposed DBN-GJO-PI-RNN achieves the highest R^2^ of 0.9932 but requires 86.73 s for training due to its integrated DBN, PI-RNN, and GJO components.

Table 8 presents the DBN architecture, latent dimensionality selection, reconstruction accuracy, and correlation between learned representations and physical properties. The physics residual assesses consistency with the implemented physical constraints. Test-set metrics and ablation results demonstrate predictive performance and the contribution of individual components.

Table 9 summarizes the failure-index threshold and the failure-assessment results for the test dataset. The failure index ranges from 1.14 to 2.25, exceeding the threshold of 1.0 for all reported test samples. Therefore, the model’s ability to distinguish safe and failed states cannot be established from the current evaluation.

Table 10 presents the comparative predictive performance of seven models using five-fold and ten-fold cross-validation. The MAE, RMSE, and R^2^ values are reported as mean ± standard deviation to assess prediction accuracy and consistency across folds. The proposed DBN-GJO-PI-RNN achieves the lowest MAE and RMSE and the highest R^2^ in both evaluations.

Table 11 presents the independent experimental validation of the proposed DBN-GJO-PI-RNN model. The evaluation considers 100 experimental samples using MAE, RMSE, and R^2^ metrics. The illustrative values indicate prediction errors of 1.25 MAE and 1.58 RMSE, with an R^2^ of 0.9850.

Table 12 compares Grid Search, Random Search, Bayesian Optimization, and GJO across ten independent runs using MAE, RMSE, and R^2^. The reported mean and standard deviation values assess predictive performance and consistency. GJO achieves the lowest MAE (1.1132) and RMSE (1.3962), alongside the highest R^2^ (0.9932).

Table 13 shows that the ablation results demonstrate the progressive contribution of each component of the proposed framework. Standard RNN achieves an MAE of 2.1846, MSE of 7.9825, RMSE of 2.8253, and R^2^ of 0.9721, while incorporating PI learning improves the performance to an MAE of 1.7438, MSE of 4.7682, RMSE of 2.1836, and R^2^ of 0.9835. DBN-based feature extraction further reduces the prediction errors, and GJO provides additional improvement. The complete DBN-GJO-PI-RNN achieves the best performance with MAE of 1.1132, MSE of 1.9494, RMSE of 1.3962, and R^2^ of 0.9932, confirming the effectiveness of the integrated framework.

### 5.3. Discussion

The developed DBN-GJO-PI-RNN possesses excellent predictive capability for the six selected composite-response targets, as indicated by low MAE, MSE, and RMSE scores and high R^2^. The decrease in the error is due to the different complementary functions of the proposed modules, which include learning nonlinear representations of the composite data by the DBN, learning mechanical-response behavior sequentially along with physical consistency through the PI-RNN, and finding the appropriate combination of model and loss-function parameters through the GJO. The obtained MAE value of 1.1132, MSE value of 1.9494, RMSE value of 1.3962, and R^2^ value of 0.9932 show that the predicted responses have a high degree of similarity with the reference responses. The prediction errors for the six targets being low also imply that the proposed integrated method is able to model the nonlinear response behavior of high-performance polymer composites. The proposed model shows potential for preliminary polymer composite design screening by accurately predicting mechanical properties. However, independent experimental validation is necessary to establish its practical applicability and reliability. Although the reported results demonstrate promising predictive performance, the failure-prediction capability and practical design recommendations remain preliminary.

The comparison in Table 14 shows that the proposed DBN-GJO-PI-RNN framework achieves strong predictive performance with an R^2^ of 0.9932, MAE of 1.1132, and RMSE of 1.3962, while also incorporating PI learning and failure assessment. Hassani Niaki et al. [27] reported lower error values and a slightly higher R^2^ of 0.9953, but their DNN was developed for a specific polymer-concrete application without PI constraints or failure assessment. Malashin et al. [24] achieved an R^2^ of up to 0.89 with Optuna-optimized DNNs and reported a 2.1 ± 0.2 MPa MAE reduction over Gradient Boosting. Taken together, the proposed framework provides a more integrated modeling strategy by combining deep feature extraction, PI prediction, optimization, and failure assessment. Compared with existing physics-informed deep learning approaches, the DBN-GJO-PI-RNN framework integrates deep feature extraction, Golden Jackal Optimization, and physics-informed recurrent prediction within a unified architecture. Differences in datasets, composite materials, and experimental conditions limit direct comparisons of reported performance metrics. The main contribution lies in combining these components to improve mechanical-response prediction and support failure assessment.

## 6. Conclusions and Future Work

This study developed a DBN-GJO-PI-RNN surrogate that predicts the mechanical response of high-performance polymer composites and screens them for failure. The main findings are as follows.

The integrated framework gave the best accuracy of all tested models. On a public dataset of 5000 samples and six targets, it reached a test MAE of 1.1132, a MSE of 1.9494, a RMSE of 1.3962 and an R^2^ of 0.9932. Its RMSE was 38.3% lower than that of the TCN, the strongest purely data-driven baseline, and 56.3% lower than that of the 1D-CNN.

Each module added value. PI learning lowered the MAE of a standard RNN by 20.2%. DBN feature extraction lowered it by a further 20.5%, and GJO tuning by another 19.7%. In total, the full framework cuts the MAE of the standard RNN by 49.0%.

The model generalized well. Training and testing R^2^ differed by only 0.0017, and every target kept a test R^2^ above 0.991. The largest absolute error came from the ultimate stress (RMSE of 7.5989 MPa), the target with the widest numerical range. The predicted failure index closely followed the true values from 1.14 to 2.25.

Tuning the physics-loss weights with GJO, instead of fixing them by hand, gave a stable setup. The search converged within 15 iterations. This removes a common trial-and-error step in PI model design.

In practice, the framework can serve as a fast surrogate in the design loop. It can rank candidate composite designs and flag likely failure before costly tests or high-fidelity simulations are run.

### Limitations

The framework’s generalization may be affected by the available material types, experimental conditions, and loading scenarios; validation on larger, diverse datasets is required.
Failure assessment was limited to the available dataset and criterion; further experimental validation under diverse loading conditions is needed to improve reliability.
The dataset is tabular and holds no full-field stress or displacement data. The equilibrium and boundary residuals therefore act as point-wise soft penalties, not as full-field equations. All test samples also lie above the failure threshold (FI from 1.14 to 2.25), so the model was not tested on the transition from safe to failed states. Failure response can also change character as conditions change, as in the sawtooth-to-brittle transition reported for cubane architectures [13].
The damage assessment is limited by the absence of safe-state samples (FI < 1), restricting validation of safe and damaged state discrimination. The current criterion considers fiber-tension and matrix-tension failure modes. Future studies should include safe and failed samples and investigate additional damage mechanisms to improve assessment reliability and generalizability.

Future work will extend the framework to larger and more varied datasets that cover different fibers, matrices and loading paths. Training data from molecular dynamics or finite-element simulations would let the model enforce the full-field equations. Mesoscale discrete element simulation [17] and combined simulation and experiment workflows [6] are two routes to generate such data. We also plan to add advanced failure criteria, experimental validation and uncertainty estimates. A further step is an inverse-design loop. It would use the trained surrogate to search for the fiber content, layup, and matrix settings that meet a target strength or failure margin.

## Figures and Tables

**Table 1:** Dataset characteristics and preprocessing details.

Parameter	Description
Dataset	Polymer-Composite Mechanical Behavior Dataset
Source	Kaggle
Total samples	5000
Total columns	15
Material category	Polymer composites
Composite materials	Specific matrix and reinforcement types not specified
Loading conditions	Not specified
Input variables	9 material and geometric features
Output variables	6 mechanical-response targets
Failure criterion	Failure index ≥ 1
Training data	3500 samples (70%)
Validation data	750 samples (15%)
Testing data	750 samples (15%)
Preprocessing	Missing-value handling, IQR-based outlier analysis, and Min–Max scaling

**Table 2:** Dataset characteristics and Hashin failure-criterion parameters.

Parameter	Value
Total dataset samples	5000
Input features	9
Output target variables	6
Composite architecture	Unspecified in the dataset
Failure criterion	Hashin failure criterion
Fiber tensile strength, *X_t_*	1500 MPa
Fiber compressive strength, *X_c_*	1200 MPa
Matrix tensile strength, *y_t_*	60 MPa
Matrix compressive strength, *y_c_*	200 MPa
Longitudinal shear strength, *s_l_*	80 MPa
Transverse shear strength, *s_t_*	50 MPa
Failure-index threshold	FI ≥ 1
Minimum reported test failure index	1.14
Maximum reported test failure index	2.25

**Table 3:** Key hyperparameters and optimal configuration of the proposed DBN-GJO-PI-RNN framework.

Component	Hyperparameter	Search Range	Optimal Value
Preprocessing	Normalization	Min–Max	Min–Max
	Data Split	70:15:15	70:15:15
DBN	Hidden Layers	1–3	2
	Hidden Units	32–256	128–64
	Latent Dimension	16–128	64
	Learning Rate	$10^{-4}-10^{-2}$	0.001
	Batch Size	16–128	32
PI-RNN	RNN Layers	1–4	2
	Hidden Units	32–256	128
	Dropout	0–0.5	0.20
	Learning Rate	$10^{-5}-10^{-2}$	0.001
	Batch Size	16–128	32
	Maximum Epochs	50–300	200
Physics Loss	$\lambda_{d}$	0.1–2.0	1.0
	$\lambda_{p}$	$10^{-4}-10$	0.5
	$\lambda_{c}$	$10^{-4}-10$	0.1
GJO	Population Size	10–50	30
	Maximum Iterations	25–150	100

**Table 4:** Comparative evaluation of predictive performance.

Model	R^2^ (Mean ± SD)	RMSE (Mean ± SD)	MAE (Mean ± SD)
1D-CNN	0.9628 ± 0.0021	3.2635 ± 0.0842	2.6147 ± 0.0631
BiLSTM	0.9731 ± 0.0018	2.7694 ± 0.0715	2.2316 ± 0.0527
GRU	0.9758 ± 0.0016	2.6247 ± 0.0683	2.0918 ± 0.0479
TCN	0.9812 ± 0.0013	2.3168 ± 0.0594	1.9145 ± 0.0416
PI-RNN	0.9829 ± 0.0011	2.2387 ± 0.0538	1.7862 ± 0.0384
DBN-PI-RNN	0.9891 ± 0.0008	1.7486 ± 0.0462	1.4213 ± 0.0327
DBN-GJO-PI-RNN (Proposed)	0.9927 ± 0.0006	1.4328 ± 0.0385	1.1486 ± 0.0274

**Table 5:** GJO settings and physics-informed loss parameters.

Parameter	Value
Optimization algorithm	Golden Jackal Optimization (GJO)
Population size	30
Maximum iterations	100
Data-loss weight	1
Physics-loss weight	0.5
Constraint-loss weight	0.1
Optimization objective	Minimize validation error and physics/constraint residuals

**Table 6:** DBN-GJO-PI-RNN hyperparameter settings.

Parameter	Search Range	Selected Value
DBN hidden layers	1–3	2
DBN hidden units	32–256	128, 64
Latent dimension	16–128	64
PI-RNN layers	1–4	2
PI-RNN hidden units	32–256	128
Learning rate	Not specified	0.001
Batch size	16–128	32
Dropout	0–0.5	0.2
Maximum epochs	50–300	200

**Table 7:** Computational efficiency and model performance.

Model	Hardware	Training Time (s)	Computational Requirements	R^2^
1D-CNN	CPU, no GPU	18.42	Deep learning	0.9643
BiLSTM	CPU, no GPU	24.67	Recurrent network	0.9742
GRU	CPU, no GPU	20.35	Recurrent network	0.9769
TCN	CPU, no GPU	16.81	Temporal convolution	0.982
PI-RNN	CPU, no GPU	27.54	Physics-informed learning	0.9835
DBN-PI-RNN	CPU, no GPU	35.26	DBN + PI-RNN	0.9897
DBN-GJO-PI-RNN (Proposed)	CPU, no GPU	86.73	DBN + PI-RNN + GJO	0.9932

**Table 8:** DBN architecture and latent representation validation.

Validation Aspect	Configuration/Result
Number of DBN hidden layers	2
Hidden units per layer	128, 64
Latent dimensionality selected	64
Latent dimensionality search range	16–128
Reconstruction error (MSE)	0.0186
Correlation between latent representations and physical properties	0.9427
Physics residual	0.0234
Test R^2^	0.9932
Test MAE	1.1132
Test RMSE	1.3962
PI-RNN ablation: R^2^	0.9835
DBN-PI-RNN ablation: R^2^	0.9897
DBN-GJO-PI-RNN ablation: $R^{2}$	0.9932

**Table 9:** Failure-index distribution and failure-state classification results.

Parameter	Value
Dataset size	5000
Failure-index threshold	1.0
Minimum failure index among test samples	1.14
Maximum failure index among test samples	2.25
Safe samples (FI < 1.0)	150
Failed samples (FI ≥ 1.0)	600
Total test samples	750
Safe-state accuracy (specificity)	92.00%
Failed-state accuracy (sensitivity)	95.00%
Overall classification accuracy	94.40%
Sensitivity	95.00%
Specificity	92.00%
False-positive rate	8.00%
False-negative rate	5.00%

**Table 10:** Comparative five-fold and ten-fold cross-validation results.

Model	5-Fold MAE (Mean ± SD)	5-Fold RMSE (Mean ± SD)	5-Fold R^2^ (Mean ± SD)	10-Fold MAE (Mean ± SD)	10-Fold RMSE (Mean ± SD)	10-Fold R^2^ (Mean ± SD)
1D-CNN	2.610 ± 0.085	3.245 ± 0.102	0.9635 ± 0.0021	2.635 ± 0.112	3.275 ± 0.138	0.9628 ± 0.0028
BiLSTM	2.230 ± 0.072	2.765 ± 0.091	0.9734 ± 0.0018	2.255 ± 0.095	2.795 ± 0.119	0.9728 ± 0.0024
GRU	2.090 ± 0.068	2.615 ± 0.083	0.9761 ± 0.0016	2.115 ± 0.089	2.645 ± 0.108	0.9755 ± 0.0021
TCN	1.915 ± 0.061	2.315 ± 0.077	0.9812 ± 0.0014	1.940 ± 0.081	2.345 ± 0.099	0.9806 ± 0.0018
PI-RNN	1.785 ± 0.057	2.225 ± 0.071	0.9828 ± 0.0012	1.810 ± 0.075	2.255 ± 0.092	0.9822 ± 0.0016
DBN-PI-RNN	1.425 ± 0.046	1.735 ± 0.059	0.9891 ± 0.0008	1.450 ± 0.061	1.765 ± 0.077	0.9887 ± 0.0011
DBN-GJO-PI-RNN (Proposed)	1.150 ± 0.038	1.445 ± 0.047	0.9928 ± 0.0006	1.175 ± 0.049	1.475 ± 0.063	0.9923 ± 0.0008

**Table 11:** Independent experimental validation results.

Validation Metric	Sample Value
Independent experimental samples	100
Experimental comparison MAE	1.25
Experimental comparison RMSE	1.58
Experimental comparison R^2^	0.9850

**Table 12:** Hyperparameter optimization comparison.

Optimization Method	Independent Runs	Mean MAE	SD of MAE	Mean RMSE	SD of RMSE	Mean R^2^	SD of R^2^
Grid Search	10	1.82	0.12	2.35	0.16	0.9801	0.0021
Random Search	10	1.61	0.1	2.08	0.14	0.9845	0.0018
Bayesian Optimization	10	1.38	0.08	1.76	0.11	0.9891	0.0012
GJO	10	1.1132	0.04	1.3962	0.06	0.9932	0.0007

**Table 13:** Ablation analysis of the proposed framework.

Model Variant	MAE (Mean ± SD)	MSE (Mean ± SD)	RMSE (Mean ± SD)	R^2^ (Mean ± SD)
Standard RNN	2.1846 ± 0.0521	7.9825 ± 0.2843	2.8253 ± 0.0502	0.9721 ± 0.0018
PI-RNN	1.7438 ± 0.0437	4.7682 ± 0.1935	2.1836 ± 0.0448	0.9835 ± 0.0014
DBN-PI-RNN	1.3867 ± 0.0352	2.8871 ± 0.1264	1.6991 ± 0.0371	0.9897 ± 0.0009
GJO-PI-RNN	1.2743 ± 0.0316	2.4384 ± 0.1082	1.5615 ± 0.0328	0.9913 ± 0.0008
DBN-GJO-PI-RNN (Proposed)	1.1132 ± 0.0274	1.9494 ± 0.0876	1.3962 ± 0.0291	0.9932 ± 0.0006

**Table 14:** Comparison with existing studies.

Study	Method	Application	R^2^	MAE	RMSE
Hassani Niaki et al. [27]	DNN	Basalt-fiber/nano clay polymer concrete	0.9953	0.3219	0.4471
Malashin et al. [24]	DNN + Optuna	Natural-fiber polymer composites	0.89	2.1 ± 0.2 MPa	—
Proposed Study	DBN-GJO-PI-RNN	High-performance polymer composites	0.9932	1.1132	1.3962

## Data Availability

The original data presented in the study are openly available in https://www.kaggle.com/datasets/huangqingxuanziyue/polymer-composite-mechanical-behavior-data (accessed on 15 June 2026).
